# Molecular mechanism and research progress on pharmacology of traditional Chinese medicine in liver injury

**DOI:** 10.1080/13880209.2018.1517185

**Published:** 2018-12-04

**Authors:** Hong Yang Zhang, Hong Ling Wang, Guo Yue Zhong, Ji Xiao Zhu

**Affiliations:** Research Center of Traditional Chinese Medicine Resources and Minority Medicine, Jiangxi University of Traditional Chinese Medicine, Nan Chang, China

**Keywords:** Herb, hepatic, liver damage, drug discovery

## Abstract

**Context:** Liver disease is a common threat to human health, caused by a variety of factors that damage the liver. Recent studies have shown that active ingredients (for example: flavonoids, saponins, acids, phenols, and alkaloids) from Traditional Chinese Medicine (TCM) can have hepatoprotective benefits, which represents an attractive source of drug discovery for treating liver injury.

**Objective:** We reviewed recent contributions on the chemically induced liver injury, immunological liver damage, alcoholic liver injury, and drug-induced liver injury, in order to summarize the research progress in molecular mechanism and pharmacology of TCM, and provides a comprehensive overview of new TCM treatment strategies for liver disease.

**Materials and methods:** Relevant literature was obtained from scientific databases such as Pubmed, Web of Science. and CNKI databases on ethnobotany and ethnomedicines (from January 1980 to the end of May 2018). The experimental studies involving the antihepatic injury role of the active agents from TCM and the underlying mechanisms were identified. The search terms included ‘liver injury’ or ‘hepatic injury’, and ‘traditional Chinese medicine’, or ‘herb’.

**Results:** A number of studies revealed that the active ingredients of TCM exhibit potential therapeutic benefits against liver injury, while the underlying mechanisms appear to contribute to the regulation of inflammation, oxidant stress, and pro-apoptosis signaling pathways.

**Discussion and conclusions:** The insights provided in this review will help further exploration of botanical drugs in the development of liver injury therapy via study on the effective components of TCM.

## Introduction

The liver is the largest parenchymal organ in the abdominal cavity, it plays an important role in regulating physiological functions, such as digestive and excretory functions, storage of nutrients, metabolic homeostasis, synthesis of new substances, and detoxification of harmful chemicals. Therefore, liver injuries and diseases are serious health problems that threaten human health. Liver diseases have a wide range of liver pathologies, including hepatic steatosis, fatty liver, hepatitis, fibrosis, cirrhosis, and hepatocarcinoma. However, only a limited number of studies have been performed up to now on the treatment options for liver injuries and diseases. Therefore, discovering a new treatment that could safely and effectively block or reverse liver injuries and diseases remains a top priority. Liver injuries due to ingestion or exposure to chemicals and industrial toxicants pose a serious health risk. Immunological liver injury is caused by immune response, which is characterized by infiltrate of inflammatory cell, formate of inflammatory granuloma, and damage of hepatic cell cable structure. Chronic or heavy drinking can lead to alcoholic liver injury, resulting in the damage of liver function. Idiosyncratic drug reactions can be extremely severe and are not accounted for by the regular pharmacology of a drug (Tailor et al. 2015). This review briefly discusses the molecular mechanism of liver damage, in the aspects of chemical liver injury, immunological liver injury, alcoholic liver injury, and drug-induced liver injury (Zhang et al. 2017). The roles of Traditional Chinese Medicine (TCM) as the agents for antihepatic injury, the pharmacological effects of all the monomers separated from Chinese medicine, and Chinese herbs on liver injury from January 1980 to May 2018 are summarized.

## Chemical liver injury

### Carbon tetrachloride (CCl_4_)

CCl_4_, a hepatotoxin, is the oldest and most frequently used toxin for inducing hepatic injury in experimental animal studies. The underlying mechanism is that various cellular enzymes are massively released into the bloodstream during liver injury (Balogun and Ashafa 2016). Cyclooxygenase-2 (COX-2) catalyzes the synthesis of prostaglandins (PGs) to promote inflammatory response as well as increase the activation and proliferation of hepatic stellate cells (HSC), which then results in hepatic fibrosis. Cytochrome P450 (CYP450) transforms CCl_4_ into toxic metabolite, affect DNA and cell membrane, which result in the oxidative degradation of lipids and change the gene expression of liver cells. In addition, hepatic cells secrete large amount of inflammatory factors such as tumor necrosis factor α (TNF-α) to stimulate immunity-associated cells and generate large amount of cytokines. The resulting mediators then induce local inflammatory response, activate cysteine aspirate protease and lead to cellular apoptosis.

The interleukin (IL) is an important inflammatory mediator and immunomodulatory factor that can up-regulate the expression of nuclear factor κB (NF-κB), heterodimer compound activated protein transcription factor 1 (AP-1) and COX-2, stimulate the induction of phosphatase A2 (PLA2) and promote the activation of B and T lymphocyte that infiltrate endothelial cells. The transforming growth factor β (TGF-β) can activate HSC, generate large amount of extracellular matrix and result in hepatic fibrosis. NF-κB can regulate the expression of leukocyte adhesion molecules and soluble inflammatory factors. In addition, CCl_4_ generates Cl and CCl_3_ ions through phase I CYP450 metabolism, which leads to the loss of membrane integrity oxidative stress, cell membrane lipid peroxidation and ultimately degeneration and necrosis of liver cells. CCl_4_-induced liver injury may also be related to Bcl-2 family and mitochondrial pathway, which can integrate with the lipid bilayer to destroy the bilayer structure of mitochondria, activate caspase-8 and modulate mitochondrial membrane permeability. Cytochrome c (Cyt-C) is often released from mitochondria to the cytoplasm during mitochondrial injury, leading to apoptotic death. Nonetheless, the autophagy level is up-regulated in CCl_4_-induced liver injury model, which can activate the stellate cells and result in hepatic fibrosis (Wunjuntuk et al. 2016).

Numerous studies have shown that TCM and its active ingredients have better therapeutic effects on liver injury caused by CCl_4_. Chinese herbal medicine, *Paederia scandens* (Lour.) Merr (Rubiaceae) (Peng et al. 2015), *Micromeria croatica* (Pers.) Schott (Labiatae) (Vladimir-Knežević et al. 2015), *Trillium tschonoskii* (Maxim.) (Liliaceae) saponin (Wu et al. 2016), and *Dicranostiga leptopodu* (Maxim.) Fedde (Papaveraceae) (Tang et al. 2017) can prevent liver damage caused by CCl_4_. *Dicranostiga leptopodu* extracts attenuated CCl_4_-induced liver damage in mice through increasing antioxidative enzyme activity to improve mitochondrial function, enhance mitochondrial respiratory function, and balance redox state. Compounds such as phloretin (Lu et al. 2017), fucoidans (Song et al. 2017), trillin (Tan et al. 2016), and *Trillium tschonoskii* saponin (Wu et al. 2016) can antagonize the liver injury induced by CCl_4_. For instance, *T. tschonoskii* saponin could exhibit protective role in CCl_4_-induced liver injury in rats, by reducing alanine aminotransferase (ALT) and aspartate transaminase (AST) levels, reversing hepatomegaly, down-regulating inflammatory factors and inhibiting hepatocyte apoptosis.

### d-Galactosamine (d-GalN)

d-GalN is a hepatotoxic and liver-damaging drug that has been extensively used as an animal model for the induction of experimental liver failure. The induced mechanism is similar to that of CCl_4_-induced liver injury. d-GalN causes glucosamine accumulation, combines with the uridine diphosphate (UDP), induces deficiency of uridine triphosphate (UTP), and suppress the activation and amount of UDP glucose pyrophosphorylase (UDPase), which result in the metabolic inhibition of carbohydrate and phospholipids. d-GalN also suppresses the synthesis of RNA and proteins in liver cells, while increasing the loss of membrane and further lead to necrocytosis. Meanwhile, Kupffer cells and liver sinusoidal endothelial cells release cytokines that enhance the expression of various enzymes and inflammatory factors. d-GalN can also combine with specific parenchymal hepatic cells and affect its integrity. A large amount of calcium ions can damage calcium homeostasis, which subsequently leads to metabolic disorders. Apart from that, it also causes the depletion of glutathione (GSH) that prohibits carbohydrate and lipid metabolisms, induces free radicals and disturbs redox balance, releases TNF-α and induces apoptosis. Therefore, d-GalN is a hepatotoxic agent that causes panlobular focal hepatocyte necrosis, polymorphonuclear cell infiltration and macrophages enlargement, which closely resembles human viral hepatitis infection. Due to its unique propensity, d-GalN has been widely used in inflammatory liver injury models to screen for potential hepatoprotective agents (Wang et al. 2016).

Chinese herb medicine *Panax ginseng* C. A. Mey (Araliaceae) (Almajwal and Elsadek 2014), *Abrus mollis* Hance (Leguminosae) (Chen et al. 2014), wild ginseng (Kim et al. 2015), and *Nymphaea candida* C. Presl (Nymphaeaceae) (Zhao et al. 2017) can attenuate d-GalN-induced liver injury, and was shown to possess hepatoprotective activity against d-GalN-induced liver injury, whereas its bioactive constituent nicotiflorin can decrease the high levels of serum enzymatic and cytokines. In addition, compounds such as limonin (Mahmoud et al. 2014), genistein (Ganai and Husain 2016), and schisandrin A (Lu et al. 2014) can increase the activation of autophagy flux, inhibit apoptosis and enhance immunity towards liver injury.

### α-Naphthyl isothiocyanate(ANIT)

ANIT is a hepatotoxicant that been used in rodents as a model for human intrahepatic cholestasis. ANIT-induced liver damage is mediated by an ANIT-GSH conjugate, which can dissociate upon crossing the canalicular membrane, yielding free GSH and ANIT in the bile. The accumulated ANIT and GSH damage the biliary epithelial cells, and thus result in intrahepatic bile duct proliferation and inflammation of interlobular bile duct. Bile regurgitation or expansion to form surrounding inflammation and liver cells injury can decrease the secretion of cholestatic jaundice, hyperbilirubinemia, and bilifaction, while increasing the levels of chemotactic factors and adhesion molecules, mediating inflammatory cells and neutrophil granulocytes as well as aggravating the inflammatory reaction. ANIT induces hepatocellular apoptosis, upregulates caspase-9 and cytochrome c expression levels, and inhibits the proliferating cell nuclear antigen (PCNA) mRNA and protein expression. In the mice model, the expression levels of endoplasmic reticulum stress–related gene markers, including glucose-regulated protein 78 (GRP78), protein kinase R-like ER kinase (PERK), eukaryotic initiation factor 2 (eIF2), inositol-requiring enzyme-1 (IRE-1) and activating transcription factor 6 (ATF6) are increased by ANIT (Yao et al. 2016). ANIT upregulates GRP78nprotein expression and activates the phosphorylation of IRE1. Additionally, ANIT increases NF-κB/IL-6/STAT signaling, triggers NF-κB activation in a concentration-dependent manner, activates PPARα, and induces liver injury by increasing ALT and AST levels. ANIT also enhances the elevation of macrophage inflammatory protein-2 (MIP-2) through the binding to the IL8Rβ receptor on neutrophils (Fang et al. 2017). Furthermore, ANIT generates a large amount of the reactive oxygen through nicotinamide adenine dinucleotide phosphate (NADPH), oxidizes the superoxide dismutase (SOD) and subsequently blocks their activation, which triggers the inflammatory response and aggravates the cholestasis and liver necrosis (Fang et al. 2017).

Chinese herb medicine *Artemisia capillaries* Thunb. (Composite) (Sun et al. 2014), *Calculus Bovis* Bezoar. (Wu et al. 2013), and rhubarb (Zhao et al. 2009) can significantly improve the liver injury caused by ANIT. For example, both emodin and rhein compounds containing in rhubarb can markedly increase neutrophil infiltration and sinus congestion in the liver cells, increase bile flow, remove jaundice and gallbladder, as well as alleviate liver damage and necrosis. The inhibitory effect of chlorogenic acid (Tan et al. 2016), sweroside (Yang et al. 2016), dioscin (Zhang et al. 2016), gentiopicroside (Tang et al. 2016), and geniposide (Wang et al. 2017) can counteract the liver injury caused by ANIT. Geniposide prevents ANIT-induced liver injury in a dose-dependent manner and reduces basolateral bile acids uptake via repression of organic anion transporting polypeptide 2 (OATP2).

### Dimethyl nitrosamine(DMN)

Dimethyl nitrosamine is a potent hepatotoxic, carcinogenic and mutagenic agent, which has been used to induce hepatic fibrosis model. DMN can generate formaldehyde and methyl alcohol after combined with CYP2EI, which results in macromolecule damages, promotes Sinusoid capillarization, and upregulates the expression of TNF-α and IL-lβ. The combined substance also stimulates inflammatory response, increases the serum procollagen type III, collagen type IV and relative antigen and induces the expressions of α smooth muscle actin (α-SMA), TGF-β1, connective tissue growth factor (CTGF), TIMP-1 and its mRNA (Liang and Tan 2013). The TLR4/MyD88 signaling pathway that suppresses TLR4 and myeloid differentiation factor 88 (MyD88) can inhibit the translocation of NF-κB and affect the levels of iNOS, NO, IL-1, IL-6, and TNF-α. Sirt1/Nrf2 signaling pathway is critical for regulating oxidative stress. Sirtuin 1 (Sirt1) adjusts oxidative stress and regulates Nrf2. Additionally, some biological molecules including HO-1, glutathione cysteine ligase catalytic subunit (GCLC), glutathione cysteine ligase modifier subunit (GCLM), and glutathione-*S*-transferase (GST) are representing Nrf2 target genes. HO-1 catalyzes heme metabolism to scavenge free radicals, while GCLC and GCLM regulate the cellular redox state to eliminate ROS. Among the interferon regulatory factors, IRF-9 induces hepatocyte apoptosis after suffering from liver injury, by decreasing Sirt 1 level and increasing p53 level. p53 plays a key role in promoting apoptosis through the upregulation of some apoptotic proteins such as caspase-9 and caspase-3 (Zhang et al. 2016). In addition, p53 significantly increases the protein levels of tropomyosin 1, TGF-β1, drosophila mothers against decapentaplegic protein (Smad) 3, α-SMA, and their mRNA expression. p53 also suppresses the mRNA expression of Smad7, in order to increase the signal transmission of TGF-β1/Smad3, enhance the activation of HSC, increase the sedimentation of extracellular matrix (ECM), accelerate the course of hepatic fibrosis and ultimately lead to the cellular apoptosis of liver cells. Following the damage to hepatic sinusoidal endothelial cells (SECs), the apoptosis of HSCs and denaturation of SECs may trigger liver cell death (Chen et al. 2015).

Chinese herb medicine *Astragalus membranaceus* (Fisch.) Bge (Leguminosae) (Cheng et al. 2017), *Rheum palmatum* L. (Polygonaceae) (Pan et al. 2015), *Amomum villosum* Lour. (Zingiberaceae) (Wang et al. 2013) and *Centella asiatica* (L.) Urban (Umbelliferae) (Choi et al. 2016) can attenuate liver injury induced by DMN. *Centella asiatica* exhibited hepatoprotective effects through the increase of antioxidant enzymes levels, while reduces the levels of inflammatory mediators in rats with DMN-induced liver injury. Moreover, this herb significantly increases the levels of antioxidant enzymes, superoxide dismutase, glutathione peroxidase, and catalase, while decreases the levels of malondialdehyde and inflammatory mediators, including interleukin (IL)-1β, IL-2, IL-6, IL-10, IL-12, TNF-α, interferon-γ (IFN-γ), and granulocyte/macrophage colony-stimulating factor (GM-GSF). Other compounds such as zingerone (Cheong et al. 2016), tetrahydrocurcumin (Weerawatanakorn et al. 2014) and garcinol (Hung et al. 2014) have shown hepatoprotective effects *in vivo*. Oral administration of garcinol remarkably inhibited AST levels and relieved liver damage induced by DMN. Moreover, garcinol may prove to be highly effective in reducing the accumulation of ECM components and suppresses the expression of α-SMA, TGF-β1 and the phosphorylation of Smad 2 and Smad 3.

### Thioacetamide (TAA)

Thioacetamide is commonly used to induce hepatotoxicity in hepatic model. The toxic effect of TAA is attributed to its biological activity exerted through oxidase systems, particularly CYP450 2E1 and FAD monooxygenase. The bioactivation of TAA further lead to the formation of reactive metabolites (thioacetamide-*S*-dioxide), followed by thioacetamide sulphene and sulfone that covalently bind to cellular macromolecules or cellular membrane. Upon binding, TAA enhances oxidative stresses, and subsequently destroys the cellular integrity and initiates necrosis. TAA can be transformed into sulfone, and then to sulfoxide through the conversion of TAA-oxysulfide by CYP450 metabolizing enzymes. These metabolites suppress the phagocytosis of the monocyte to red blood cells, eliminate the capability of the endotoxin of Kupffer cells, promote endotoxemia formation, and lead to enterogenous toxicemia. Depletion of macrophages extends coagulation necrosis with elevated levels of hepatic enzymes at the early stage, which results in unsuccessful reparative fibrosis and dystrophic calcification at the end stage.

After the formation of intestinal endotoxemia (IETM), the endotoxin can inhibit hepatic microcirculation, down-regulate the expression of endothelial nitric oxide synthase (eNOS) and up-regulate the expression of iNOS, in order to increase the NO content of the liver homogenate, cause large-scale induction of hepatocyte necrosis and promote thrombosis formation. On the other hand, the endotoxin can affect the hepatic microcirculation via Kupffer-induced thromboxane A2 (TXA2) and leukotriene formation. The generated free radicals and reactive oxygen can irreversibly bind with liver macromolecules, and thereby contribute to oxidative stress. TAA also enhances the production of ROS and leads to lipid peroxidation, depletion of glutathione, and SH-Thiol group reduction. TAA induces the mobilization of calcium from intracellular stores. Both calcium and ROS can promote cell damage or proliferation by activating multiple mechanisms. In addition, TAA can interfere with the transfer of RNA from nucleus to cytoplasm, which linked to the membrane injury. Consequently, the elevated liver enzymes (ALT, AST) are the indicators for cellular liver necrosis (Khattab et al. 2017). The oxidative stress generated by TAA may also decrease the level of endogenous antioxidant such as α-tocopherol. Besides, TAA can affect the synthesis of proteins and enzyme activity, induce DNA damage and cause liver apoptosis (Zhou and Zhao 2013).

Chinese herb medicine *Antrodia cinnamomea* (Polyporaceae) (Chen et al. 2014), *Andrographis paniculata* (Burm. f.) Nees (Acanthaceae) (Bardi et al. 2014) and *Salvia miltiorrhiza* Bge. (Labiatae) (Parajuli et al. 2015) can significantly ameliorate liver injury induced by TAA. The standardized fraction of *Salvia miltiorrhiza*, PF2401-SF’s showed anti-fibrotic effects in the TAA model, through reduced HSC activation, and may be mediated by downregulation of collagen 1(α), tissue inhibitor of metalloproteinases 1 (TIMP1) and α-SMA. The bioactive compounds such as osthole (Liu et al. 2015), genistein (Dalia et al. 2014), and epigallocatechin gallate (Jamal et al. 2015) can be used to treat liver injury induced by TAA. For instance, epigallocatechin gallate administration before TAA-induced liver injury may down-regulates the expression levels of uncoupling protein 2 (UCP2), IL-6, IL-18, TNF-α, and C-reactive protein (CRP).

## Immunological liver injury

### Concanavalin A (ConA)

Concanavalin A is a plant lectin, mainly mediated by CD4 + T lymphocyte and natural killer T cells. ConA-induced liver injury model demonstrated better simulation on the pathogenesis of human immune disease and immunological liver injury mechanism. Autoimmune liver injury induced by ConA displays a time-based feature, in which the most severe liver lesions occurred at 8 h after ConA treatment (Wang et al. 2017). ConA is absorbed into the splenic organ in order to activate the proliferation of T lymphocytes (e.g Th1 and Th2) and secrete Thl and Th2 cytokines. Large amount of cytokines including TNF-α, IFN-γ, IL-2, IL-1β, etc. enter the liver through portal vein and combine with the macrophage to activate CD4 + T, mitogen of T lymphocytes, and Kupffer cells. Subsequently, NF-κB and IRF-3 are activated to induce the expression of proinflammatory cytokine and adhesion molecules, generate various inflammatory cytokines, injure liver cells, and cause damage to vascular endothelial cells, lymphocytes, monocytes, and Kupffer cells (Zheng et al. 2016). Upon the activation and upregulation of NKT cells, a variety of cytokines such as IFN-γ and IL-4 are rapidly secreted. NKT cells can directly cause liver injury through Fas/Fas ligand mechanism that secretes various cytokines to recruit and activate other innate immune cells for exacerbating inflammatory responses in the liver (Wei et al. 2016). ConA expresses major histocompatibility complex (MHC) class II structures and stimulates Kupffer cells to secrete cytokines, TNF-a and IL-1b, which subsequently activating CD4 + T cells that initiate an inflammatory reaction. After ConA administration, autophagy is initiated to decompose damages on SECs. Autophagy contributes to the transport of proteins from the cytoplasm into the lumen of antigen-processing compartments in Kupffer cells, which enhances MHC class II-mediated antigen presentation and prolongs CD4 + T cell activation. The MHCII-dependent activation of CD4+ helper T cells secrete IFN-g and further activate Kupffer cells to form a positive feedback loop. The IFN-γ can enhance ConA-induced endothelial cell autophagy and accelerate cell death (Chen et al. 2016).

Chinese herb medicine such as *Hypericum perforatum* L. (Oleaceae) (Li et al. 2015), *Angelica sinensis* (Oliv.) Diels (Umbelliferae) (Wang et al. 2016), and *Dracocephalum heterophyllum* Benth. (Labiatae) (Zheng et al. 2017) may attenuate the liver injury caused by ConA. Pretreatment with *Dracocephalum heterophyllum* extract ameliorates liver injury and suppresses the production of inflammatory cytokines (e.g TNF-α and IFN-γ) in ConA-induced hepatitis model. This herbal extract recruites CD11b + Gr1+ myeloid-derived suppressor cells to the liver and causes suppression of macrophage infiltration. Other TCM compounds such as quercetin (Li et al. 2016), shikonin (Liu et al. 2016), fucoidan (Li et al. 2016), and astaxanthin (Li et al. 2015) can be used to treat liver injury induced by ConA. Astaxanthin can attenuate serum liver enzymes and pathological damage by reducing the release of inflammatory factors. It also exhibited anti-apoptotic effects via decreasing the phosphorylation of β-cell lymphoma-2 (Bcl-2) downregulated by JNK/p-JNK pathway.

### Bacillus calmette guerin (BCG) combined with lipopolysaccharide (LPS)

The combination of Bacillus calmette guerin and lipopolysaccharide may result in acute liver injury, which is widely used as a model to study the pathogenesis of viral hepatitis and select liver-protective drugs through immunization route. The pathological changes and liver injury mechanisms are similar to that of viral hepatitis type B. Tissue damage is caused by the liver necrosis and large amount of monocyte infiltration, which is characterized by the presence of granuloma in the liver cells. This is an allergic reaction caused by cell-mediated immunity. BCG attenuates the activated TB-PCR vaccine, by triggering and sensitizing T lymphocytes and increasing the number of Kupffer cells, macrophages, and neutrophil granulocyte in the liver, in order to remain the allergic state (Liu 1991). After treatment with LPS, the allergic Kupffer cells and macrophages become activated to release a large number of cytotoxic factors such as TNF-α, NO, IL, free radicals, and leukotrienes, which ultimately resulted in hepatic necrosis. NO is a major intercellular and intracellular signaling molecule, which is essential in maintaining homeostasis and acts as a cytoprotective mediator or apoptosis inducer. NO induces vasodilatation, inhibits platelet aggregation and reduces hepatic damage. The BCG/LPS-induced immunological liver injury is an important model for pathophysiological course in sepsis and systemic inflammatory syndrome with a massive release of both NO and inducible nitric oxide synthase (iNOS). Excess production of NO from iNOS-mediated hepatotoxicity has the potential to induce various cytokines. The reaction between NO and superoxide anions may produce peroxynitrite, which the latter is a potent cytotoxic oxidative agent that elicits cellular damage (Gound et al. 2015).

TCM including *Inula britannica* L. (Composite) (Hong et al. 2012), *Atractylodes macrocephala* Koidz. (Composite) (Zhang et al. 2016), *Cichorum glandulosum* Boiss. (Composite) (Xin et al. 2014) have therapeutic effect on the experimental animal models. *C. glandulosum* significantly increases the activity of CAT, SOD, GSH-PX, and decreases the level of NO, NO synthase, hydroxyproline, ALP and lipid peroxidation in the BDG + LPS model. This herb attenuates hepatic inflammation via downregulating TNF-α, IL-6, and TGF-β as well as inhibiting protein expression of CYP2E1, NF-κB, p-ERK1/2, and COX-2, while induces the expression of p-P38 MAPK. Several compounds from *Cordyceps sinensis* (Berk.) Sacc (Clavicepitaceae) such as corn peptides (Guo et al. 2009), Atractylenolide I (Wang et al. 2012) and mycelial polysaccharides (Dong et al. 2016) may reduce the serum ALT, AST and NO levels while significantly decrease the expression levels of IL-1β, IL-6, TNF-α, and inducible nitric oxide synthase (iNOS) in hepatic tissues.

### d-GalN combined with LPS

The combination of d-GalN with LPS-induced liver injury model is commonly used to study endotoxic liver injury. d-GalN combines with UDP of liver cells, depletes UTP, suppresses the synthesis of RNA and proteins, and results in the metabolic blockage and cell death. LPS can directly activate Toll-like receptor (TLR) 4 on the surface of HSC as well as NF-JB and ERK pathways, suppress the expression of receptors and induce the expression of inflammatory cytokines and adhesive factors (Cui et al. 2016). In addition, it can also activate the expression of TNF-α, IL-6, and NO, and damage the structural integrity of the endothelial cells in response to inflammatory mediators. LPS activates Kupffer and inflammatory cells to secrete various inflammatory cytokines, including TNF-α, IL-1β, COX-2, etc. After confronted with LPS/TNF-α stimulation, Sirt1 may deacetylate p65 and compromise NF-κB activity in hepatocytes, leading to the increased susceptibility to endotoxemic injury. Among them, TNF-α is the most important pleiotropic cytokine, which can trigger an inflammatory cascade to induce cytokines, such as IL-1β, IL-6, NO, and cell adhesion molecules (Liao et al. 2017). The binding of TNF to its receptor on the hepatocyte membrane may activate caspase-3, stimulate the expressions of endothelial cells, promote the formation of thrombus and induce hepatocyte apoptosis or necrosis in acute liver failure. In addition, the combination of d-GalN and LPS can cause damage to intracellular DNA of the liver cells, by breaking into 80–200 bp or other oligonucleotide probe sections. It also activates the proteins and pathways related to the apoptosis of endoplasmic reticulum stress, induces CHOP, caspase-12, and C-Jun/JUK, as well as increasing the serum and hepatic tissue levels of TNF-α, the liver caspase-3 activity and mitochondrial pathway, in order to induce cell death (Yan et al. 2016).

TCM such as *Cistanche tubulosa* (Schenk) R.Wright (Orobanchaceae) (Lee et al. 2017), *Ceratonia siliqua* L. (Caesalpiniaceae) and *Cordyceps sinensis* (Berk.) Sacc (Clavicipitaceae) (Cheng et al. 2014) can ameliorate the liver injury induced by d-GalN/LPS. The cordycepin isolated from *C sinensis* can inhibit hepatic neutrophil and macrophage infiltration as well as prevent proinflammatory cytokine production through the suppression of TLR4 and NF-κB signaling transduction. The blockage of lipid peroxidation and reactive oxygen species (ROS) production by cordycepin is associated with the reduction of NAD(P)H oxidase activity, which significantly prevents the excessive autophagy induced by GalN/LPS in the liver. TCM compounds such as morin (Tian et al. 2017), cordycepin (Gao et al. 2016; Li et al. 2017), and mangiferin (Pan et al. 2016) can inhibit hepatic injury induced by GalN/LPS. Mangiferin protects against GalN/LPS-induced liver injury by activating the Nrf2 pathway and inhibiting NACHT, LRR, and PYD domains-containing protein 3 (NLRP3) inflammasome activation. Mangiferin also inhibits the levels of serum ALT, AST, IL-1β, TNF-α, monocyte chemotactic protein 1 (MCP-1), RANTES, hepatic malondialdehyde (MDA), ROS, d-GalN/LPS-induced hepatic NLRP3, ASC, and caspase-1 expression, while upregulate the expression of nuclear factor erythroid 2 p45 related factor 2 (Nrf2) and heme oxygenase 1 (HO-1).

### Alcoholic liver injury

The alcoholic liver injury is defined as the hepatocytic damage due to excessive intake of alcohol for long-term. In human, alcohol dehydrogenase 2 (ADH2) and aldehyde dehydrogenase-2 (ALDH2) are the two important enzymes that can eliminate the alcohol found primarily in the liver. ADH2 can catalyze the conversion of ethyl alcohol into acetaldehyde, while ALDH2 is involved in the oxidation of acetaldehyde to acetic acid. The acetaldehyde can induce oxidative stress and injure the mitochondria and microtubule system. The ethyl alcohol can generate ROS through CYP 2E1 metabolism, trigger mitochondria dysfunction, induce mitochondrial permeability transition pore and result in the release of mitochondria apoptotic factors such as cytochrome c and Smac. The release of cytochrome c promotes the activation of caspase-9 and caspase-3, whereas Smac inhibits X-linked inhibitor of apoptosis protein (XIAP) to abolish its inhibition on caspases, which then leads to hepatocyte apoptosis. The long-term ethyl alcohol exposure may alter the mitochondrial structure and function, suppress the oxidative phosphorylation and tricarboxylic acid cycle, decrease the amount of ATP and GSH as well as increase ROS (Wang et al. 2016). Therefore, mitochondrial DNA damage and the impaired synthesis of mitochondrial proteins may trigger liver injuries. In addition, long-term overconsumption of alcohol activates Kupffer cells to generate large amount of NF-κB, which promotes the release of TNF-α, IL-1, and other inflammatory cytokines. TNF-a binds to its tumor necrosis factor receptor 1 (TNFR1) and recruits tumor necrosis factor receptor-related domain death protein (TRADD), fas-associating protein with a novel death domain (FADD), caspase-8, and FLIPL. As a result, caspase-8 is activated when receptor-interacting protein (RIP) 1 is de-ubiquitinated by cylindromatosis (CYLD). Activation of caspase-8 cleaves Bid to trigger the mitochondrial apoptotic pathway and cell death. Activated caspase-8 also cleaves RIP1 and RIP3 to prevent the unleashing of necroptosis mediated by RIP1 and RIP3. RIP1 and RIP3 interact with each other via RHIM domains to form the amyloid-like necrosome, during the depletion of cIAPs and inhibition of caspase-8. The auto- and transphosphorylated RIP1 and RIP3 further recruit and phosphorylate downstream mixed lineage kinase domain-like pseudokinase (MLKL) to initiate necroptosis. Thus alcohol overconsumption may initiate a series of molecular events that can lead to the fatty degeneration, inflammation, and necrosis, stimulate the liver cell proliferation, suppress the activation of collagenase and promote the formation of liver fibrosis (Souli et al. 2015).

Chinese herb medicine of *Ceratonia siliqua* (Neyrinck et al. 2015), *Lindera aggregata* (Sims) Kosterm. (Lauraceae) (Wang et al. 2016) and Crude Rhubarb (Yang et al. 2015) have been used to improve the alcoholic liver injury. Rhubarb contained the highest amount of anthraquinones that can reduce the liver inflammation induced by alcohol, promote the growth of gut bacterium *Akkermansia muciniphila* and regulate colon antibacterial protein secretion. Several bioactive compounds such as salvianolic acid B (Zhang et al. 2017), dihydroartemisinin (Xu et al. 2017), and *Panax notoginseng* saponins (Zhou et al. 2015) can inhibit hepatic injury induced by alcohol. *Panax notoginseng* saponins inhibit acute ethanol-induced liver injury, by reducing lipolysis in white adipose tissue and decreases the expression of hormone-sensitive lipase total protein and its phosphorylation. Moreover, this herb inhibits alcohol-induced hepatic fatty acid uptake by elevating liver cluster of differentiation 36 (CD36) expressions, improves liver lipid accumulation, reduces CYP2E1-mediated oxidative stress, and prevents induction of CYP2E1 in response to chronic alcohol consumption.

## Drug-induced liver injury

### Acetaminophen (APAP)

Acetaminophen-induced acute liver injury model is commonly used to test drugs through the depletion of hepatic GSH in liver injury. A large dose of APAP can be oxidized into *N*-acetylbenzoquinoneimine (NABQI) through the combination of CYP450 and GSH, to produce bromphenyl-acetyl-cysteine or cysteine. Meantime, the remaining *N*-acetyl-*p*-benzoquinonimine (NAPQI) can have complexation with perssads containing electrons and impaired mitochondrial functions, by generating superoxide anions and hydrogen peroxide, stimulating oxidative stress reaction, increasing iNOS, and NO synthesis. Consequently, these reactions may initiate lipid peroxidation, protein oxidation, and nitration, alter the calcium homeostasis, decrease the synthesis of ATP, release miR-122 from hepatocytes, and regulate Notch signaling (Jiang et al. 2017; Singh et al. 2017). APAP aggravates the oxidative damage to mitochondrial functions and changes in membrane permeability to increase the level of superoxide. However, mitophagy was shown to improve toxicity from APAP, by clearing the damaged mitochondria and reduced the production of free radicals and ROS. APAP can result in the formation of autophagosomes that engulfed mitochondria. SQSTM1/p62, an autophagy receptor protein, was recruited to APAP adducts, which may be removed by the induction of autophagy. Therefore, timely removal of adducts via autophagy may promote protection from APAP, by increasing the expression of TNF-α, activating NF-KB p65, inflammatory factors and JNK signaling pathways. In APAP experimental model, the JNK/Sab/Src/ROS pathway sustains JNK activation, amplifies mitochondrial ROS production and promotes MPT-mediated necrosis. For apoptosis, this pathway also sustains elevated levels of JNK activation and modulated multiple antiapoptotic Bcl2 family members that mediate mitochondrial outer membrane permeabilization. In fact, cell death in APAP model is a form of regulated necrosis, which is mediated by MPT and ultimately leads to liver cells death (Dara and Kaplowitz 2017).

TCM *Dicranopteris linearis* (Burm.) Underw. (Adiantaceae) (Zakaria et al. 2017), *Cymbopogon citratus* (DC.) Stapf. (Gramineae) (Uchida et al. 2017), *Panax ginseng* (Hu et al. 2017), *Apocynum venetum* L. (Apocynaceae) (Xie et al. 2015) can improve the liver injury caused by APAP. For instance, *A. venetum* inhibited the activation of caspase-3, cleavage of DNA, released of cytochrome C and significantly reduced serum aminotransferase, MDA, and 3-nitrotyrosine (3-NT) levels, which exhibited a protective effect against liver injury. Furthermore, compounds such as hyperoside (Xie et al. 2016), baicalin (Liao et al. 2017), astaxanthin (Zhang et al. 2017), and resveratrol (Wang et al. 2015) have therapeutic effect on liver injury induced by APAP. For example, resveratrol can inhibit the activation of APAP into toxic metabolites NAPQI, inhibit APAP-induced JNK activation, negatively regulate p53 signaling to induce cell proliferation-associated proteins including cyclin D1, cyclin-dependent kinase 4 (CDK4), proliferating cell nuclear antigen (PCNA), and sirtuin type 1 (SIRT1); thus promote the proliferation of liver cells.

### Isoniazid (INH) combined with rifampin (RIF)

The combined use of Isoniazid and rifampin hepatotoxic drugs is a common treatment for tuberculosis. INH is primarily metabolized in the liver through acetylation process, catalyzed by *N*-acetyl transferase 2 enzyme. INH is acetylated into acetyl-isoniazid (AcINH), which can be oxidized further into acethydrazide, and subsequently hydrolyzed into hydrazine by covalently binding with biomacromolecule. AcINH is then metabolized to form toxic monoacetyl hydrazine (MAH), lesser toxic diacetyl hydrazine (DAH) and other minor metabolites of INH. The CYP450 superfamily further metabolize INH intermediates through phase I pathways of drug metabolism. MAH and its reactive metabolites cause liver injury possibly through free radical generation. The production of reactive oxygen species enhances the extent of lipid peroxidation, thereby causing damage to the cellular membrane of hepatocytes. Depletion of GSH can result in lipid peroxidation of cytomembrane and mitochondrial membrane, change the permeability of mitochondrial membrane and lead to liver damage. RIF reacts through deacetylation in liver, in order to donate acetyl group for the acetylation of INH, induction of CYP450 and accelerate the metabolism of AcINH into acethydrazide. Hence, RIF is thought to potentiate the formation of toxic INH metabolites, by enhancing hepatic idiosyncratic drug reactions in the liver cells. This combination of drugs can down-regulate the expressions of peroxisome proliferator-activated receptors (PPAR) α mRNA and Bsep mRNA. As a result, these drugs have been reported to cause necrosis/cell injury, inflammation, hepatic steatosis induce cholestasis by impairing bile secretion, increase the consumption of hepatic glycogen, affect the microcirculation of liver, and ultimately lead to cell death and destroy endoplasmic reticulum (Baskaran and Sabina 2015).

*Ficus religiosa* L. (Moraceae) (Parameswari et al. 2013), *Bacopa monnieri* L. (Composite) (Evan et al. 2016), *Tamarix gallica* L. (Tamaricaceae) (Urfi et al. 2018), monoammonium glycyrrhizin (Zhou et al. 2016) and kaempferol (Shih et al. 2013) can reduce the depletion of glutathione and MDA levels, and thus inhibit hepatic injury induced by INF/RIF.

## Conclusions

Necrosis and apoptosis induced by liver injury exist in almost all liver diseases, but the mechanism of triggering liver injury and apoptosis is specific. This is mainly due to the combined effect of the process of metabolism and detoxification associated with hepatotoxic drugs or components in the liver and the imbalance between different degrees of injury in liver cell and cell protection pathway (For example: apoptosis, innate immune response, repair, and regeneration, etc). Although Chinese medicine has a certain effect, its ingredients are numerous, and the effective components are still not clear. Therefore, the more definite pathogenesis and mechanism of liver protection is the key and difficult points of today’s research. This process is very complex and miscellaneous and requires the cooperation of experts in various fields to further study and promote clinical treatment and drug development.

### Strengthen the synergy of TCM research

TCM and its effective components can improve liver functions, reduce injury caused by oxidative stress, regulate immunologic functions, decrease inflammation, and apoptosis, as well as protect against liver damage. These studies lay the foundation for future development of hepatoprotective drugs. However, the basic concept of TCM is the effective compound groups, especially herbal medicine that has large group of chemical components. Therefore, the study on TCM drug action cannot be limited to one or several effective constituents but is relying on the exact mechanisms. Moreover, the use of the synergistic approach in TCM is needed for disease prevention, diagnosis, and treatment.

### The synergistic effects of the same and different targets

Because of the different pathogenic mechanisms in the development of liver injury, multiple targets are involved in different liver injury models. The current challenge is to identify the key target and index model. The target selection should be based on the conceptual foundation of pharmacology, by establishing theoretical models of TCM and thorough assessment of the target and related biology. Thus, the safety and efficiency of TCM can be improved. Although there are several studies on the multiple targets of TCM in the treatment of liver injury, further studies on the synergistic effects of the effective components of TCM with the same target are needed. It is proposed that different components of TCM with the same target and the synergistic effects of different targets may be the possible mechanisms of effective TCM compounds. Therefore, more fundamental studies should be conducted on the functions of different constituents for the key target as well as the synergistic effects of same and different targets, in order to identify the mechanisms underlying potential therapeutic role of TCM.

### Highlight the combination of the basis with clinical applications

By understanding the molecular mechanisms underlying liver injury and pharmacological effects of TCM, the discovery and development of new TCM with potential clinical applications can be improved. However, there are numerous basic fundamental researches been carried out, but clinical studies are lacking. In future, the combined efforts of basic, translational, and clinical studies should be highlighted, in the context of treatment combination. The beneficial values of the combined therapy between Chinese and Western medicines should be exploited, in order to develop an effective and inexpensive drug for the treatment of liver injuries.

## Figures and Tables

**Table 1.** Comparison of various types of liver injury. (A summary and comparison of all kind of major models experimental models of liver injury, including type, model, key index, molecular mechanism, advantages, and disadvantages.)

Type	Model	Key index	Molecular mechanism	Advantages	Disadvantages
CCl_4_	40%–50% CCl_4_ vegetable oil solution	ALT, AST, TNF-α, TLR, TGF-β, IL, NF-κB	1. Inflammation 2. Oxidative stress 3. Hepatic fibrosis 4. Mitochondrial pathway 5. Apoptosis	1. Good repeatability 2. Economics	1. High mortality 2. Poisonous
d-GalN	d-GalN solution	ALT, AST、 GSH, TNF-α, TLR, IL, NF-κB	1. Inflammation 2. Oxidative stress 3. Metabolic disorder 4. Necrosis 5. Apoptosis	1. Does not affect other organs 2. A better model for evaluating therapeutic efficacy of viral hepatitis	Expensive
ANIT	ANIT olive oil solution	ALT, AST, SOD, GSH, IL, NADPH	1. Inflammation 2. Hepatotoxicity 3. Cholestasis Necrosis	1. Simple and easy 2. Good reproducibility	Expensive
DMN	1% DMN solution	ALT, AST, TNF-α, TLR, TGF-β, α-SMA	1. Inflammation 2. Hepatic fibrosis 3. Apoptosis	1. A good model for studying hepatic fibrosis 2. The rate of hepatic fibrosis is high and relatively stable after formation	1. More toxic 2. Volatile Excreta contain poisons and pollute the environment
TAA	TAA solution	ALT, AST, NO, SOD, TNF-α, IL, NF-κB	1. Metabolic disorder 2. Enterogenous toxicemia 3. Apoptosis 4. Necrosis	1. Low mortality 2. High success rate	Expensive
ConA	ConA solution	ALT, AST, TNF-α, IL, NF-κB, NLRP3	1. Inflammation 2. Apoptosis 3. Necrosis	1. Simple operation 2. No pre sensitization is required	There is no viral replication and sustained injury to the liver parenchyma
BCG/LPS	BCG/LPS normal saline solution	ALT, AST, NO, TNF-α, TGF-β, IL, NF-κB	1. Sensitization 2. Hepatocellular damage	1. Short molding time 2. Simple and convenient	Pre-sensitized with BCG
d-GalN/LPS	d-GalN/LPS solution	ALT, AST, NO, TNF-α, TGF-β, IL, NF-κB	1. Metabolic block 2. Protein synthesis 3. Hepatocyte DNA fracture and degradation 4. Necrosis	It is a good model for endotoxin-induced liver injury	Pre-sensitized with d-GalN
Alcoholic	White spirit	ALT, AST, SOD, TNF-α, IL, GSH, NF-κB	1. Inflammation 2. Oxidative stress 3. Hepatic fibrosis Mitochondrial 4. Pathway 5. Necrosis 6. Apoptosis	Stable and reliable	1. Long molding time 2. Frequent gastric lavage is more complicated
APAP	APAP normal saline solution	ALT, AST, NO, SOD, TNF-α, IL, GSH, NF-κB	1. Inflammation 2. JNK pathway 3. Necrosis	Very sensitive to mice	Insensitive to rats
INH/RIF	INH/RIF solution	ALT, AST, GSH, MDA	1. Inflammation 2. Cholestasis 3. Metabolic disorder 4. Necrosis	Stable	Expensive

**Table 2.** A summary of the effect of traditional Chinese medicine on liver injury. (This is an overview of liver injury treated by Chinese medicine by the end of May 2018, including the name of herb or compound, parts or solvent, liver injury model, mechanism of action, and reference.)

Name of herb or compound	Parts or Solvent	Liver injury model	Experiment	Mechanism of action	Reference
*Aconitum carmichaelii* Debeaux	Aqueous extract	d-Gal-N	vivo	Inhibited inflammatory reaction, apoptosis and promoted liver tissue regeneration in the acute liver failure rats.	Luo J et al. 2016. Aqueous extract from *Aconitum carmichaelii* Debeaux reduces liver injury in rats via regulation of HMGB1/TLR4/ NF-ΚB/ caspase-3 and PCNA signaling pathways. J Ethnopharmacol.
*Acremoniumterricola milleretal* mycelium	Aqueous extract	CCl_4_	vivo	Scavenge free radicals, decrease the level of TGF-β1 and block TGF-β/Smad signaling pathway.	Tian X et al. 2011. Effects and Mechanisms of *Acremoniumterricola milleretal* mycelium on Liver Fibrosis Induced by Carbon Tetrachloride in Rats. The American Journal of Chinese Medicine.
*Aesculus hippocastanum*	Aqueous extract	ConA	vivo	Significantly protect the Con A-induced acute liver injury in mice via inhibition of ROS and JNK pathway.	Wu SJ et al. 2017. The hepatoprotective effect of *Aesculus hippocastanum* seed extract against concanavalin A-induced acute liver injury in mice via inhibition of ROS and JNK pathway. Chinese Pharmacological Bulletin
*Antrodia camphorate* Zang & Su	Methanol extract	LPS	vivo	Utilize the NF-κB and MAPKs pathways and the regulation of SOD activity to attenuate LPS-induced nonspecific pulmonary inflammation	Huang GJ et. 2014. Methanol extract of *Antrodia camphorata* protects against lipopolysaccharide-induced acute lung injury by suppressing NF-κB and MAPK pathways in mice. Journal of Agricultural and Food Chemistry.
Antrosterol	*Antrodia camphorata* synonym	CCl_4_	vivo	Inhibited lipid peroxidation, enhance the activities of antioxidant enzymes, decreases the TNF-α level, NO and iNOS, and COX-2 expressions.	Huang G et al. 2012. Protective effect of antrosterol from *Antrodia camphorata* submerged whole broth against carbon tetrachloride-induced acute liver injury in mice. Food Chemistry.
*Anoectochilus formosanus* Hay. and *Gynostemma pentaphyllum* Makino	Methanol extract and aqueous extract	APAP	vivo	Increased levels of AST and ALT	Lin C et al. 2000. Antioxidant and hepatoprotective effects of * Anoectochilus formosanus* and *Gynostemma pentaphyllum*. The American Journal of Chinese Medicine.
*Arctium- lappa* L.	Aqueous extract	APAP and CCl_4_	vivo	Decrease the GSH, MDA, and CYP450	Lin SC et al. 2000. Hepatoprotective effects of *Arctium lappa* on carbon tetrachloride- and acetaminophen-induced liver damage. The American Journal of Chinese Medicine.
*Artemisia capillaris* Thunb	Aqueous extract	CCl_4_	vivo	Successfully lower serum ALT and AST activities, restore the GSH level, ameliorate or restore GPx and CAT activities as well.	Yang C C et al. 2012. Potential antioxidant properties and hepatoprotective effects of an aqueous extract formula derived from three Chinese medicinal herbs against CCl_4_-induced liver injury in rats. International Immunopharmacology.
Chlorogenic acid	*Lonicera japonica* Thunb.	APAP	vitro (L-02 cells)	Reversed the decreased cell viability; reduced serum levels of ALT/AST; prevented liver oxidative stress injury.	Chun P et al. 2015. Chlorogenic acid prevents acetaminophen-induced liver injury: the involvement of CYP450 metabolic enzymes and some antioxidant signals. J Zhejiang Univ Sci B.
**Cirsium setidens** Nakai	n-butanol extract	CCl_4_	vivo	Transcript levels of genes encoding antioxidant enzymes such as GPO1, GPO3 and SOD1 were elevated.	Lee SH et al. 2008. Antioxidant and Hepatoprotective Activities of *Cirsium setidens* NAKAI against CCl_4_-Induced Liver Damage. American Journal of Chinese Medicine.
Esculentoside A	*Phytolacca esculen-ta* van Houtte	CCl_4_ and _D_-GalN/LPS	vivo	Attenuated CCl_4_ and _D_-GalN/LPS-induced acute liver injury in mice and its protective effects might be involved in inhibiting inflammatory response and oxidative stress.	Zhang F et al. 2014. The protective effect of esculentoside an on experimental acute liver injury in mice. PLoS ONE.
Esculin	*Cortex Fraxini* Bark	LPS	vivo	Diminished the protein expression of NF-κB p65 in liver, which resulted in lower levels of inflammatory mediators.	Li W et al. 2016. Esculin attenuates endotoxin shock induced by lipopolysaccharide in mouse and NO production in vitro through inhibition of NF-κB activation. Eur J Pharmacol.
*Ferulago campestris* Besser	Essential oil	_D_-GalN/LPS	vivo	Decreased oxidative stress and inhibiting cytokines.	Li W et al. 2016. The Chemical Constituents and the Hepato-protective Effect of the Essential Oil of *Ferulago campestris* (Besser) Grecescu (Apiaceae). Journal of Essential Oil Bearing Plants.
Gallic acid	*Rhus chinensis* Mill.	CCl_4_	vivo	Protective effect of these substances on cell membranes.	Kanai S et al. Mechanism of the protective effects of sumac gall extract and gallic acid on the progression of CCl_4_-induced acute liver injury in rats. The American Journal of Chinese Medicine.
Gentiopicroside	*Gentiana scabra* Bunge	ANIT	vivo	Change bile acids metabolism which highlights its importance in mitigating cholestasis, resulting in the marked decrease of intracellular bile acid pool back toward basal levels.	Tang X et al. 2016. Target profiling analyses of bile acids in the evaluation of hepatoprotective effect of gentiopicroside on ANIT-induced cholestatic liver injury in mice. J Ethnopharmacol.
Glycyrrhiza uralensis Fisch.	*Polysaccharide*	CCl_4_	vivo	Decrease of NOS and NO levels, and reduction of the production of free radicals	Chen D et al. 2016. Study on Protective Effect of Glycyrrhiza uralensis *Polysaccharide* on CCl_4_ Induced Acute Liver Injury in Mice and Its Mechanism. China Pharmacy.
Glycyrrhiza uralensis Fisch.	*Total saponins*	CCl_4_	vivo	Decreased in serum ALT and AST	Gao XY et al. 2011. Preparation of total licorice saponin and research on its hepatoprotective effect. Pharmacology and Clinics of Chinese Materia Medica.
Homoplantaginin	*Salvia plebeia* R. Br	BCG/LPS	vitro (HL-7702 cells)	Decreased in serum ALT and AST, decreased the levels of TNF-α and IL-1, elevated the levels of GSH, GSH-Px and SOD.	Qu XJ et al. 2009. Protective effects of *Salvia plebeia* compound homoplantaginin on hepatocyte injury. Food and Chemical Toxicology.
*Hypericum japonicum* Thunb.	Aqueous extract	ANIT and CCl_4_	vivo	Had an obvious effect on the decreasing of AST, ALT and T-BIL levels in serum.	Wang N et al. 2008. Hepatoprotective effect of *Hypericum japonicum* extract and its fractions. Journal of Ethnopharmacology.
Isofuranodiene	*Curcua zedoara* Christ. Rosc	_D_-GalN/LPS	vivo	Significantly inhibited _D_-GalN/LPS-induced mRNA expression of IL-1β, IL-6, and iNOS in liver tissues.	Chen XP et al. 2015. Isofuranodiene protect D-galactosamin/lipopolysacchride induced liver injury in rat**s.** Chin J Pharm Toxicol.
Kaerophyllin	*Bupleurum chinense* DC.	TAA	vivo	Suppressed hepatic inflammation and inhibiting HSC activation, possibly through upregulation of PPAR-γ expression.	Lee T et al. 2012. Protective effects of kaerophyllin against liver fibrogenesis in rats. European Journal of Clinical Investigation.
Kukoamine B	*cortex Lycii*	LPS	vivo	Inhibited inflammation in septic mice by reducing the concentrations of plasma LPS, decreased leukocyte sequestration and interfering with NF-κB activation, suppressed the pro-adhesive phenotype of endothelial cells.	Qin W et al. 2015. A novel role of kukoamine B: Inhibition of the inflammatory response in the livers of lipopolysaccharide-induced septic mice via its unique property of combining with lipopolysaccharide. Exp Ther Med.
*Litsea coreana* Levl.	total flavonoids	CCl_4_	vivo	Inhibited the expression of TGF-β1 and increased the expression of PPAR-γ.	Huang C et al. 2010. Potential protective effects of a traditional Chinese herb, *Litsea coreana* Levl., on liver fibrosis in rats. Journal of Pharmacy and Pharmacology.
*Lxeris chinensis* Thunb.	Aqueous extract	CCl_4_	vivo	Inhibited the acute elevation of serum transaminases.	Lin SC et al. 1994. Hepatoprotective effects of Taiwan folk medicine: *Ixeris chinensis* (Thunb.) Nak. on experimental liver injuries. The American Journal of Chinese Medicine.
Magnolol	*Magnolia officinalis* Rehd.	ConA	Vitro (Th17 Cells)	Inhibited Th17 cell differentiation; suppressed IL-17A generation.	Zhang H et al. 2017. Magnolol Attenuates Concanavalin A-induced Hepatic Fibrosis, Inhibits CD4+ T Helper 17 (Th17) Cell Differentiation and Suppresses Hepatic Stellate Cell Activation: Blockade of Smad3/Smad4 Signalling. Basic & Clinical Pharmacology & Toxicology.
Matrine	*Sophora flavescens* Ait	CCl_4_	vivo	Prevented monocyte infiltration into the injured livers and inhibition of MCP-1 production and activity.	Shi D. et al. 2013. Matrine Inhibits Infiltration of the Inflammatory Monocyte Subset in Injured Mouse Liver through Inhibition of Monocyte Chemoattractant Protein-1. Evidence-based Complementary and Alternative Medicine.
*Oenanthe javanica* Blume	total phenolics	_D_-Gal-N	vivo	Improve the survival of acute liver failure model significantly and prevent the elevation of the serum enzymatic markers and nonenzymatic markers levels significantly.	Guo A et al. 2016. The protective effect of total phenolics from *Oenanthe javanica* on acute liver failure induced by d-Galactosamine. J Ethnopharmacol.
Oridonin	*Rabdosia rubescens(Hamst.)* Y. wuet. Hsuan	_D_-Gal-N/LPS	vivo	Attenuated _D_-GalN/LPS-induced apoptosis in hepatocytes by reducing pro-apoptotic signals.	Deng Y et. 2017. Oridonin ameliorates lipopolysaccharide/D-galactosamine-induced acute liver injury in mice via inhibition of apoptosis. Am J Transl Res.
*Orychophragmus violaceus*	Aqueous extract	CCl_4_	vivo	Prevent CCl_4_-induced liver injury in mice via regulating the Nrf2 and NFκB pathways.	Huo X et al. 2017. Hepatoprotective Effect of Aqueous Extract from the Seeds of *Orychophragmus violaceus* against Liver Injury in Mice and HepG2 Cells. Int J Mol Sci.
Osthole	*Cnidium monnieric* (L.) Cuss.	APAP	vivo	Inhibited the metabolic activation of APAP and enhanced its clearance through an antioxidation mechanism.	Cai Y et al. 2018. Osthole prevents acetaminophen-induced liver injury in mice. Acta Pharmacol Sin.
*Paeonialactiflora* Pall. And *Astragalus membranaceus* Fisch.	70% aqueous ethanol	BCG/LPS	vivo	Reduced nitric oxide production and suppressed Kupffer cell activity and pro-inflammatory mediator and cytokines production.	Sun W et al. 2008. Protective effect of extract from *Paeonia lactiflora* and *Astragalus membranaceus* against liver injury induced by bacillus Calmette-Guérin and lipopolysaccharide in mice. Basic and Clinical Pharmacology and Toxicology.
*Panax ginseng* C. A. Mey	Aqueous extract	CCl_4_	vivo	Induced oxidative stress in rats	Karadeniz A et al. 2009. Protective effect of *Panax ginseng* on carbon tetrachloride-induced liver, heart and kidney injury in rats. Revue de Medicine Veterinaire.
*Penthorum chinense* Pursh	Aqueous extract	CCl_4_	vivo	Ameliorated CCl_4_-induced oxidative stress via activating Nrf2 signaling pathway.	Wang M et al. 2017. Hepatoprotective properties of *Penthorum chinense* Pursh against carbon tetrachloride-induced acute liver injury in mice. Chin Med.
*Penthorum chinense* Purs	Aqueous extract	Alcoholic	vivo	Suppressed CYP2E1-mediated oxidative stress and enhancing the oxidant defense systems via the activation of Nrf2/HO-1 pathway.	Cao Y et al. 2015. Protective effects of *Penthorum chinense* Pursh against chronic ethanol-induced liver injury in mice. Journal of Ethnopharmacology.
Periplocoside A	*Periploca sepium* Bge	ConA	vivo	Inhibited of NKT-derived inflammatory cytokine productions.	Wan J et al. 2008. Periplocoside A, a pregnane glycoside from *Periploca sepium* Bge, prevents concanavalin A-induced mice hepatitis through inhibiting NKT-derived inflammatory cytokine productions. International immunopharmacology.
*Pogostone*	*Pogostemon cablin* (Blanco) Benth	LPS	vivo	Suppressed the production of the proinflammatory mediators in serum, and attenuate liver injury.	Lia Y. 2014. Pogostone suppresses proinflammatory mediator production and protects against endotoxic shock in mice. Journal of Ethnopharmacology.
*Polygonatum sibiricum*	*Polysaccharides*	CCl_4_	vivo	Reduce the activities of ALT, AST, ALP and the contents of DBIL, TBIL in serum of rats.	Han CY et al. 2018. Study on extraction of *Polygonatum sibiricum* polysaccharides and its protective effect on CCl_4_-induced acute liver injury in rats. Acta Agriculturae Zhejiangensis
puerarin	*Radix Puerariae* Lobatae	Alcoholic and CCl_4_	vivo	Mediates hepatoprotection by the inhibition of inflammatory response and downregulation of the TNFα/NFκB pathway	Zhu P L et al. 2015, Effects of combined dietary supplementation with fenofibrate and Schisandrae Fructus pulp on lipid and glucose levels and liver function in normal and hypercholesterolemic mice. Drug Design, Development and Therapy
puerarin	*Radix Puerariae* Lobatae	_D_-Gal-N/LPS	vivo	Increase autophagy and suppress of apoptosis	Li L et al. 2018. Protective role of puerarin on LPS/D-Gal induced acute liver injury via restoring autophagy. Am J Transl Res.
*Radix Polygoni* Multiflori	Methanol extract	DMN	vivo	Reverse the pathogenic progression of the disease, decrease the hydroxyproline content and increases the expression of HGF messenger RNA in liver tissue	Huang C et al. 2007. Chinese herb *Radix Polygoni* Multiflori as a therapeutic drug for liver cirrhosis in mice. Journal of Ethnopharmacology.
*Rheum Palmatum* L.	total extract; 90% ethanol	CCl_4_	vivo	Liver toxicity of High dose, liver preservation of low dose.	Wang JB et al. 2011. Hepatotoxicity or Hepatoprotection? Pattern Recognition for the Paradoxical Effect of the Chinese Herb *Rheum palmatum* L. in Treating Rat Liver Injury. PLoS ONE.
*Rhus chinensis* Mill.	Aqueous extract	CCl_4_	vivo	Protective effect of these substances on cell membranes.	Kanai S et al. Mechanism of the protective effects of sumac gall extract and gallic acid on the progression of CCl_4_-induced acute liver injury in rats. The American Journal of Chinese Medicine.
Saikosaponin-d	*Bupleurum chinense* DC.	DMN	vivo	Reduced collagen I deposition in the liver and ALT level in the serum; decreased the content of TGF-1 in the liver.	Fan J et al. 2007. Saikosaponin-d attenuates the development of liver fibrosis by preventing hepatocyte injury. Biochemistry and Cell Biology.
Saikosaponin-d	*Bupleurum chinense* DC.	CCl_4_	vivo	Alleviated hepatocyte injury from oxidative stress.	Fan J et al. 2007. Saikosaponin-d attenuates the development of liver fibrosis by preventing hepatocyte injury. Biochemistry and Cell Biology.
Saikosaponin-d	*Bupleurum chinense* DC.	APAP	vivo	Down-regulating NF-κB- and STAT3-mediated inflammatory signaling.	Aiming L et al. 2014. Saikosaponin d protects against acetaminophen-induced hepatotoxicity by inhibiting NF-κB and STAT3 signaling. Chemico-Biological Interactions.
*Salvia miltiorrhiza* Bge.	Polysaccharide	BCG/LPS	vivo	Effectively improved the liver index, spleen index and thymus index; reduced the serum levels of ALT, AST and NO; restored liver homogenate contents of TNF-α and IL-1β.	Song YH et al. 2008. Protection of a polysaccharide from *Salvia miltiorrhiza*, a Chinese medicinal herb, against immunological liver injury in mice. International Journal of Biological Macromolecules.
*Salvia miltiorrhiza* Bunge.	Aqueous extract	CCl_4_	vivo	Reduced levels of transforming growth factor-beta1, procollagens I and III and tissue inhibitor of metalloproteinase-1 transcripts and an increased level of matrix metalloproteinase-13 transcript.	Wasser S et al. 1998. *Salvia miltiorrhiza* reduces experimentally-induced hepatic fibrosis in rats. Journal of Hepatology.
Salvianolic acid-A	*Salvia miltiorrhiza* Bge.	CCl_4_	vivo	Have protective effects against liver injury and fibrosis in rats.	Hu Y Y et al. 2000. Protective actions of salvianolic acid A on hepatocyte injured by peroxidation in vitro. World Journal of Gastroenterology.
*Schisandra chinensi-s* （Turcz.）Baill	Aqueous extract	CCl_4_	vivo and clinical	Induced liver cytochrome P-450 and promote certain anabolic metabolism such as serum protein biosynthesis and glycogenesis.	Liu GT. 1989. Pharmacological actions and clinical use of fructus schizandrae. Chin Med J (Engl).
Schisandrin B	*Schisandra chinensis* Baill	d-Gal-N	vivo	Proteins 27 and 70 were involved in the protective effect.	Gao Z et al. 2016. Heat shock proteins 27 and 70 contribute to the protection of Schisandrin B against d-galactosamine-induced liver injury in mice. Canadian Journal of Physiology and Pharmacology.
Scutellarin	*Scutellaria baicalensis* Georgi	Con A	vivo	Inhibited the NF-kappaB-TNF-α-iNOS transduction pathway.	Zheng H et al. 2007. The protective action of scutellarin against immunological liver injury induced by concanavalin A and its effect on pro-inflammatory cytokines in mice. Journal of Pharmacy and Pharmacology.
*Semen Persicae* and *Flos Carthami*	Aqueous extract	CCl_4_	vivo	Inhibited pathological angiogenesis and hepatic fibrogenesis may be through affecting the angiogenesis-associated VEGF and its upstream and downstream signaling pathways.	Xi S et al. 2016. The Effects of Taoren-Honghua Herb Pair on Pathological Microvessel and Angiogenesis-Associated Signaling Pathway in Mice Model of CCl_4_-Induced Chronic Liver Disease. Evidence-Based Complementary and Alternative Medicine.
Shikonin	*Lithospermum erythrorhizon* Sieb.	d-Gal-N/LPS	vivo	Inhibited TLR4 signaling pathway	Lin MX et al. 2017. Shikonin protects against D-Galactosamine and lipopolysaccharide-induced acute hepatic injury by inhibiting TLR4 signaling pathway. Oncotarget.
Silymarin	*Silybum marianum* (L.) Gaertn.	CCl_4_	vivo	Reduce ALT elevation in animals exposed to CCl_4_.	Li Y et al. 2003. Traditional Chinese Medicine Prevents Inflammation in CCI_4_-Related Liver Injury in Mice. American Journal of Chinese Medicine.
Sparstolonin B	*Scirpus yagara* Ohwi	LPS	vivo	Suppressed LPS-induced increase of TNF-α and IL-6 in serum and livers.	Liang QL et al. 2015. Protective effects of Sparstolonin B, a selective TLR2, and TLR4 antagonist, on mouse endotoxin shock. Cytokine.
*Stephania tetrandra* Moore. and *Salvia miltiorrhiza* Bge.	Aqueous extract	CCl_4_	vivo	Safely and effectively prevents and reverses hepatic fibrosis through activating HSC apoptosis in rats	Josette S Y et. 2009. *Stephania tetrandra* prevents and regresses liver fibrosis induced by carbon tetrachloride in rats. Journal of gastroenterology and hepatology.
Tannins	*Toxicodendron vernicifluum* (S-tokes)	CCl_4_	vivo	The mechanism for this prevention might be due mainly to the protective effect of these substances on cell membranes.	Kanai S et al. 1998. Mechanism of the protective effects of sumac gall extract and gallic acid on the progression of CCl_4_-induced acute liver injury in rats. The American Journal of Chinese Medicine.
Thaliporphine	*Thalictrum aquilegifolium* L.	LPS	vivo	Suppressed TNF-α, NO, and O^2-^ production.	Chiao C et al. 2005. Thaliporphine increases survival rate and attenuates multiple organ injury in LPS-induced endotoxaemia. Naunyn Schmiedeberg's Archives of Pharmacology.
Tetrandrine	*Tephania tetrandra* S. Moore	ConA	vivo	Suppressed the production of various inflammatory mediators in the liver through inhibited of NF-κB activation.	Feng D et al. 2008. Tetrandrine protects mice from concanavalin A-induced hepatitis through inhibiting NF-κB activation. Immunology Letters.
*Wedelia chinensis (Osbeck)* Merr.	Aqueous extract	CCl_4_, APAP, _D_-Gal-N	vivo and clinical	Improve the general damage condition and suppress enzyme leakage from cellular membranes.	Lin SC et al. 1994. Hepatoprotective effects of Taiwan folk medicine: *wedelia chinensis* on three hepatotoxin-induced hepatotoxicity. The American Journal of Chinese Medicine.

**Figure 1.**
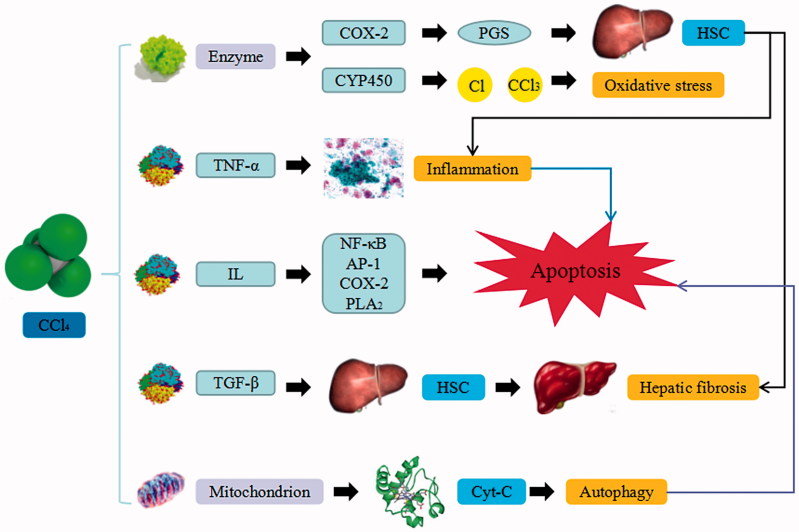
CCl_4_-induced liver injury. CCl_4_ trigger the continuous development of hepatocyte injury by inducing cell stress via inflammation, macrophages activation, mitochondrion path, and oxidative stress, resulting in apoptosis, hepatic fibrosis, and liver injury. COX-2: epoxide hydrolase; PGs: prostaglandins; HSC: hepatic stellate cells; CYP450: cytochrome P450; TNF-α: tumor necrosis factor α; IL: interleukin; NF-κB: nuclear factor κB; AP-1: activated protein transcription factor 1; PLA_2_: phosphatase A2; TGF-β: transforming growth factor β; Cyt-C: cytochrome C.

**Figure 2.**
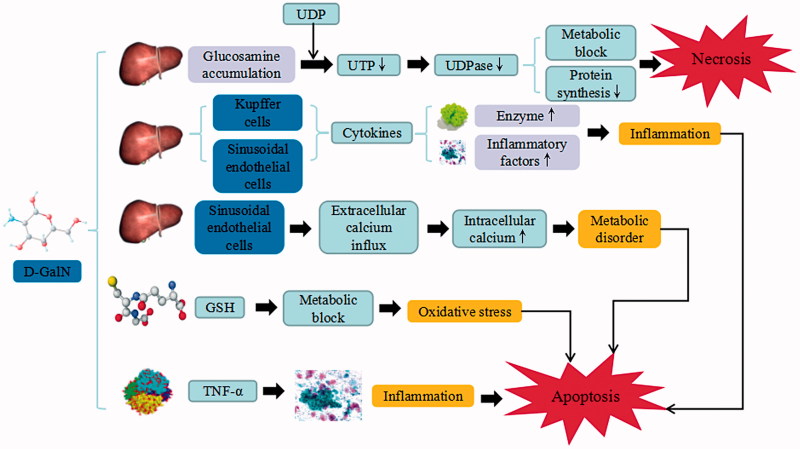
d-GalN-induced liver injury. d-GalN trigger the continuous development of hepatocyte injury by inducing cell stress via inflammation, metabolic disorder, and oxidative stress, resulting in necrosis, apoptosis, autophagy, and liver injury. UDP: uridine diphosphate; UTP: uridine triphosphate; UDPase: UDP-glucose pyrophosphorylase; GSH: glutathione; TNF-α: tumor necrosis factor α.

**Figure 3.**
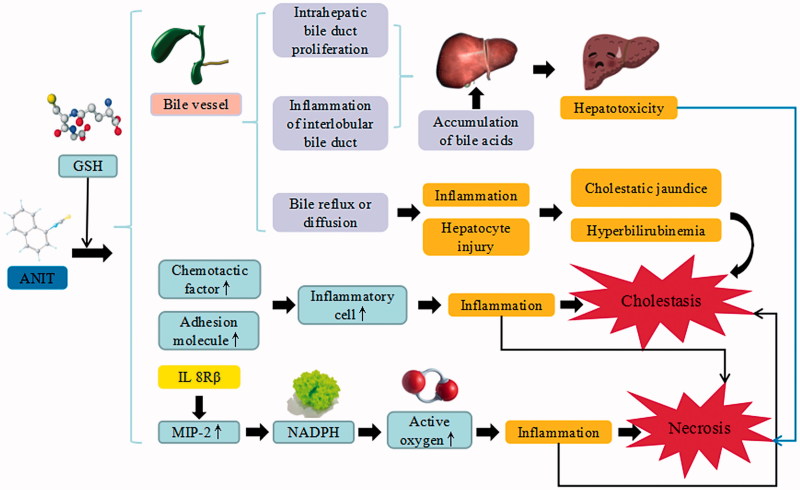
ANIT-induced liver injury. ANIT trigger the continuous development of hepatocyte injury by inducing cell stress via inflammation, hepatotoxicity, cholestatic jaundice, and hyperbilirubinemia, resulting in apoptosis, autophagy, cholestasis, and liver injury. GSH: glutathione; IL: interleukin; MIP-2: macrophage inflammatory protein-2; NADPH: nicotinamide adenine dinucleotide phosphate.

**Figure 4.**
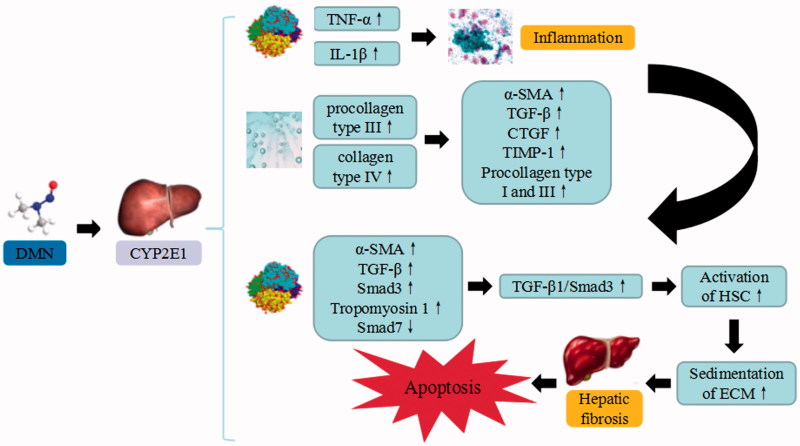
DMN-induced liver injury. DMN trigger the continuous development of hepatocyte injury by inducing cell stress via inflammation, and activation of HSC, resulting in apoptosis, autophagy, sedimentation of ECM, hepatic fibrosis, and liver injury. CYP2EI: cytochrome P2EI; TNF-α: tumor necrosis factor α; IL: interleukin; α-SMA: α smooth muscle actin; TGF-β: transforming growth factor β; CTGF: connective tissue growth factor; TIMP1: tissue inhibitor of metalloproteinases 1; Smad: drosophila mothers against decapentaplegic protein; ECM: extracellular matrix.

**Figure 5.**
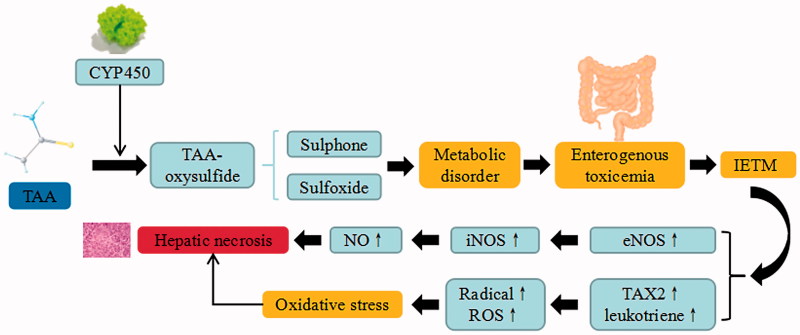
TAA-induced liver injury. TAA trigger the continuous development of hepatocyte injury by inducing cell stress via inflammation, metabolic disorder, enterogenous toxicemia, and oxidative stress, resulting in hepatic necrosis, apoptosis, and liver injury. CYP450: cytochrome P450; IETM: intestinal endotoxemia; iNOS: inducible nitric oxide synthase; eNOS: endothelial nitric oxide synthase; TXA_2_: thromboxane A_2_; ROS: reactive oxygen species.

**Figure 6.**
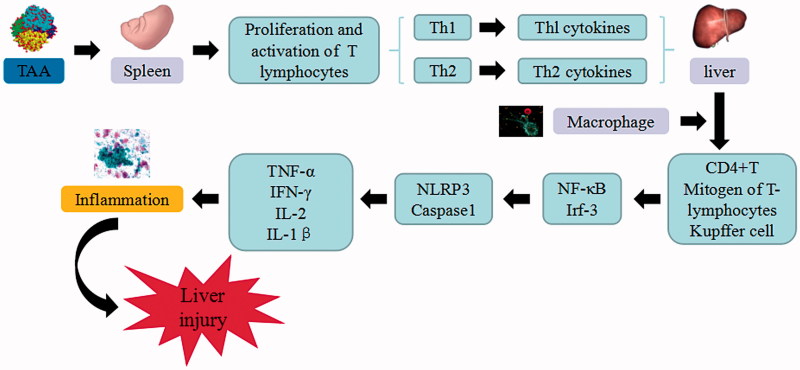
ConA-induced liver injury. ConA triggers the continuous development of hepatocyte injury by inducing cell stress via inflammation and macrophages activation, resulting in hepatic necrosis and liver injury. NF-κB: nuclear factor κB; Irf-3: Interferon regulatory factor -3; NLRP3: NACHT, LRR and PYD domains-containing protein 3; TNF-α: tumor necrosis factor α; IFN-γ: Interferon-γ; IL: interleukin.

**Figure 7.**
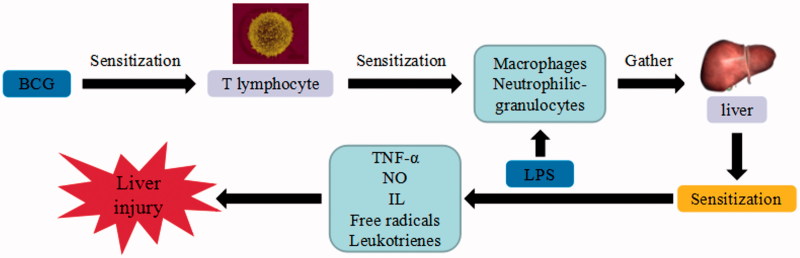
BCG/LPS-induced liver injury. BCG/LPS trigger the continuous development of hepatocyte injury by inducing cell stress via inflammation and sensitization, resulting in hepatic necrosis and liver injury. TNF-α: tumor necrosis factor α; IL: interleukin.

**Figure 8.**
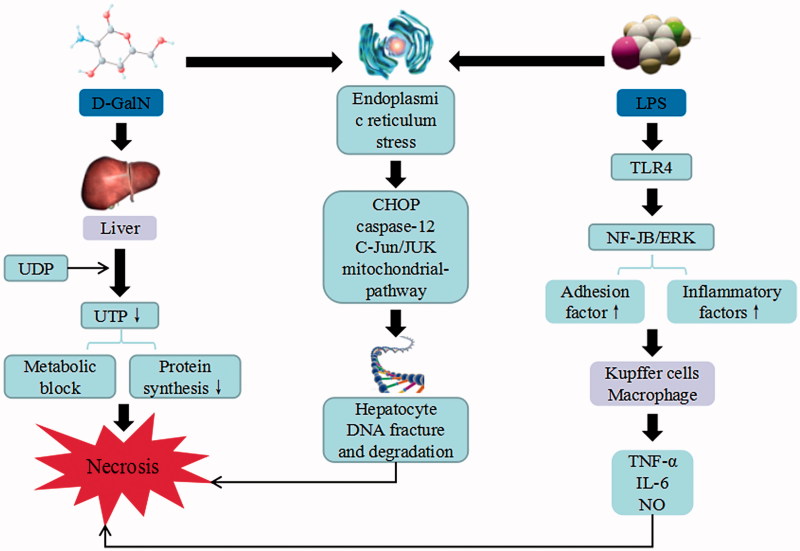
d-GalN/LPS-induced liver injury. d-GalN/LPS triggers the continuous development of hepatocyte injury by inducing cell stress via inflammation, metabolic block, protein synthesis decrease, and hepatocyte DNA fracture and degradation, resulting in hepatic necrosis and liver injury. UDP: uridine diphosphate; UTP: uridine triphosphate; TLR: toll-like receptor; TNF-α: tumor necrosis factor α; IL: interleukin.

**Figure 9.**
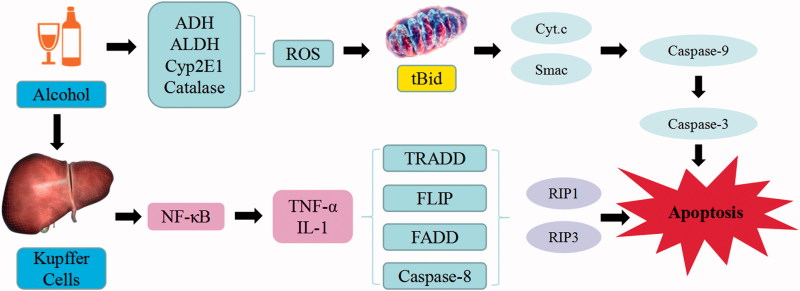
Alcoholic-induced liver injury. Alcohol triggers the continuous development of hepatocyte injury by inducing cell stress via inflammation, oxidative stress, protein synthesis decrease, and hepatocyte DNA fracture and degradation, resulting in hepatic necrosis, apoptosis, hepatic fibrosis, and liver injury. ADH: alcohol dehydrogenase; ALDH: aldehyde dehydrogenase; CYP2EI: cytochrome P2EI; ROS: reactive oxygen species; Cyt-C: cytochrome C; RIP: receptor-interacting protein; TRADD: tumor necrosis factor receptor-related domain death protein; FADD: fas-associating protein with a novel death domain; TNF-α: tumor necrosis factor α; IL: interleukin; NF-κB: nuclear factor κB.

**Figure 10.**
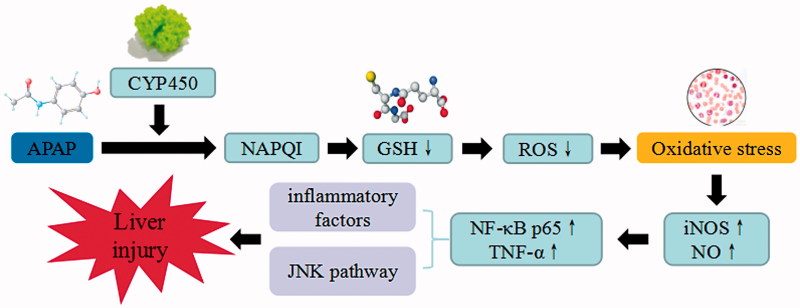
APAP-induced liver injury. APAP trigger the continuous development of hepatocyte injury by inducing cell stress via inflammation, oxidative stress, and JNK pathway, resulting in hepatic necrosis, apoptosis, hepatic fibrosis, and liver injury. CYP450: cytochrome P450; NAPQI: *N*-acetyl-*p*-benzoquinonimine; GSH: glutathione; ROS: reactive oxygen species; iNOS: inducible nitric oxide synthase; NF-κB: nuclear factor κB; TNF-α: tumor necrosis factor α.

**Figure 11.**
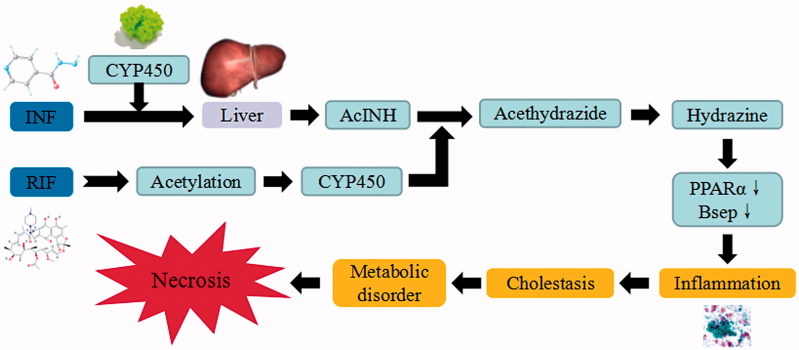
INH/RIF-induced liver injury. INH/RIF trigger the continuous development of hepatocyte injury by inducing cell stress via inflammation, cholestasis, and metabolic disorder, resulting in hepatic necrosis, apoptosis, and liver injury. CYP450: cytochrome P450; PPAR: peroxisome proliferator-activated receptors.

## References

[CIT0001] Almajwal A, Elsadek MF 2014 Anti-hepatotoxic prospect of *Panax ginseng* extract and/or selenium against d-galactosamine-induced liver injury in experimental rats. Prog Nutr. 16:16–24.

[CIT0002] Balogun FO, Ashafa AOT 2016 Antioxidant, hepatoprotective and ameliorative potentials of aqueous leaf extract of *Gazania krebsiana* (Less.) against carbon tetrachloride (CCl_4_)-induced liver injury in Wistar rats. Trans R Soc S Afr. 71:145–156.

[CIT0003] Bardi DA, Halabi MF, Hassandarvish P, Rouhollahi E, Paydar M, Moghadamtousi SZ, Al-Wajeeh NS, Ablat A, Abdullah NA, Abdulla MA 2014 *Andrographis paniculata* leaf extract prevents thioacetamide-induced liver cirrhosis in rats. PLoS One. 9:e109424. 25280007 10.1371/journal.pone.0109424 PMC4184875

[CIT0004] Baskaran UL, Sabina EP 2015 The food supplement coenzyme Q10 and suppression of antitubercular drug-induced hepatic injury in rats: the role of antioxidant defence system, anti-inflammatory cytokine IL-10. Cell Biol Toxicol. 31:211–219. 26374116 10.1007/s10565-015-9305-x

[CIT0005] Chen YR, Chang KT, Tsai MJ, Lee CH, Huang KJ, Cheng H, Ho YP, Chen JC, Yang HH, Weng CF 2014 *Antrodia cinnamomea* profoundly exalted the reversion of activated hepatic stellate cells by the alteration of cellular proteins. Food Chem Toxicol. 69:150–162. 24751970 10.1016/j.fct.2014.04.006

[CIT0006] Chen M, Wang T, Jiang ZZ, Shan C, Wang H, Wu MJ, Zhang S, Zhang Y, Zhang LY 2014 Anti-inflammatory and hepatoprotective effects of total flavonoid C-glycosides from *Abrus mollis* extracts. Chin J Nat Med. 12:590–598. 25156284 10.1016/S1875-5364(14)60090-X

[CIT0009] Chen K, Li J, Li S, Feng J, Wu L, Liu T, Zhang R, Xu S, Cheng K, Zhou Y, et al. 2016 15d-PGJ2 alleviates ConA-induced acute liver injury in mice by up-regulating HO-1 and reducing hepatic cell autophagy. Biomed Pharmacother. 80:183–192. 27133055 10.1016/j.biopha.2016.03.012

[CIT0007] Cheng YJ, Cheng SM, Teng YH, Shyu WC, Chen HL, Lee SD 2014 *Cordyceps sinensis* prevents apoptosis in mouse liver with d-galactosamine/lipopolysaccharide-induced fulminant hepatic failure. Am J Chin Med. 42:427–441. 24707872 10.1142/S0192415X14500281

[CIT0008] Cheng Y, Mai JY, Wang MF, Chen GF, Ping J 2017 Antifi brotic effect of total flavonoids of *Astmgali Radix* on dimethylnitrosamine-induced liver cirrhosis in rats. Chin J Integrative Med. 23:48. 10.1007/s11655-016-2627-6 27787720

[CIT0010] Chen GF, Ping J, Gao YQ, Jin SG 2015 Effects of dimethylnitrosamine on acute toxic liver injury in different age and gender mice. Chin J Inte Trad West Med Liver Dis. 25:92–94.

[CIT0011] Cheong KO, Shin DS, Bak J, Lee C, Kim KW, Je NK, Chung HY, Yoon S, Moon JO 2016 Hepatoprotective effects of zingerone on carbon tetrachloride and dimethylnitrosamine-induced liver injuries in rats. Arch Pharmacal Res. 39:279–291. 10.1007/s12272-015-0696-2 26667466

[CIT0012] Choi MJ, Zheng HM, Kim JM, Lee KW, Park YH, Lee DH 2016 Protective effects of *Centella asiatica* leaf extract on dimethylnitrosamine-induced liver injury in rats. Mol Med Rep. 14:4521–4528. 27748812 10.3892/mmr.2016.5809 PMC5101987

[CIT0013] Cui X, Chen Q, Dong Z, Xu L, Lu T, Li D, Zhang J, Zhang M, Xia Q 2016 Inactivation of Sirt1 in mouse livers protects against endotoxemic liver injury by acetylating and activating NF-κB. Cell Death Dis. 7:e2403–e2407. 27711079 10.1038/cddis.2016.270 PMC5133964

[CIT0014] Dalia OS, Gehad ARAJ, Sally AEA, Fatma O, Manal B 2014 Thioacetamide-induced liver injury: protective role of genistein. Can J Physiol Pharmacol. 92:965–973. 25358106 10.1139/cjpp-2014-0192

[CIT0015] Dara L, Kaplowitz N 2017 Cell death in drug-induced liver injury. Springer Int Pub. 18:1018–1053. 10.3390/ijms18051018 PMC5454931 28486401

[CIT0016] Dong KZ, Gao YS, Wang XH, Ma YQ, Su L 2016 Protective effect of mycelial polysaccharides from *Cordyceps sinensis* on immunological liver injury in mice. Med J Chin PLA. 41:284–288.

[CIT0017] Evan PS, Udhaya LB, Sunitha PS, Geetha A 2016 Reparation of isoniazid and rifampicin combinatorial therapy-induced hepatotoxic effects by *Bacopa monnieri*. Pharmacol. 98:29–34. 10.1159/000444856 27007136

[CIT0018] Fang ZZ, Tanaka N, Lu D, Jiang CT, Zhang WH, Zhang CZ, Du Z, Fu ZW, Gao P, Cao YF 2017 Role of the lipid-regulated NF-κB/IL-6/STAT3 axis in alpha-naphthyl isothiocyanate-induced liver injury. Arch Toxicol. 91:2235–2244. 27853831 10.1007/s00204-016-1877-6 PMC6331015

[CIT0019] Ganai AA, Husain M 2016 Genistein attenuates d-GalN induced liver fibrosis/chronic liver damage in rats by blocking the TGF-b/Smad signaling pathways. Chem Biol Int. 261:80–85. 10.1016/j.cbi.2016.11.022 27876602

[CIT0020] Gao YZ, Zhao LF, Ma J, Ma J, Xue WH, Zhao H 2016 Protective mechanisms of wogonoside against lipopolysaccharide/d-galactosamine-induced acute liver injury in mice. Eur J Pharmacol. 780:8–15. 26921756 10.1016/j.ejphar.2016.02.040

[CIT0021] Gound SS, Thakare VN, Khan S, Wadekar RR, Naik SR 2015 Ameliorative effects of *Tricholepis glaberrima* in experimentally induced hepatic damage in rats: modulation of cytokines functions. Journal of Ethnopharmacology. 160:164–172. 25479155 10.1016/j.jep.2014.11.037

[CIT0022] Guo H, Sun J, He H, Yu GC, Du J 2009 Antihepatotoxic effect of corn peptides against *Bacillus* calmette-guerin/lipopolysaccharide-induced liver injury in mice. Food Chem Toxicol. 47:2431–2435. 19577609 10.1016/j.fct.2009.06.041

[CIT0023] Hong T, Dong M, Gao Y, Zhao J, Wu Y 2012 Hepato-protective effect of polysaccharides extracted from *Inula britannica* flower for mice. Chem Res Chin Univ. 28:1026–1030.

[CIT0025] Hu JN, Liu Z, Wang Z, Li XD, Zhang LX, Li W, Wang YP 2017 Ameliorative effects and possible molecular mechanism of action of black ginseng (*Panax ginseng*) on acetaminophen-mediated liver injury. Mol. 22:664–672. 10.3390/molecules22040664 PMC6154718 28430162

[CIT0026] Hung WL, Tsai ML, Sun PP, Tsai CY, Yang CC, Ho CT, Cheng AC, Pan MH 2014 Protective effects of garcinol on dimethylnitrosamine-induced liver fibrosis in rats. Food Function. 5:2883–2891. 25183344 10.1039/c4fo00342j

[CIT0027] Jamal MH, Ali H, Dashti A, Al-Abbad J, Dashti H, Mathew C, Al-Ali W, Asfar S 2015 Effect of epigallocatechin gallate on uncoupling protein 2 in acute liver injury. Int J Clin Exp Pathol. 8:649–654. 25755758 PMC4348928

[CIT0028] Jiang LF, Ke M, Yue S, Xiao W, Yan YD, Deng XZ, Ying QL, Li J, Ke BB 2017 Blockade of notch signaling promotes acetaminophen-induced liver injury. Immunol Res. 65:739–749. 28286920 10.1007/s12026-017-8913-3 PMC5464368

[CIT0029] Khattab A, Hassanin L, Zaki N 2017 Self-nanoemulsifying drug delivery system of coenzyme (Q10) with improved dissolution, bioavailability, and protective efficiency on liver fibrosis. Aaps PharmSciTech. 18:1657–1672. 27677262 10.1208/s12249-016-0632-x

[CIT0030] Kim SJ, Choi HS, Cho HI, Jin YW, Lee EK, Ahn JY, Lee SM 2015 Protective effect of wild Ginseng cambial meristematic cells on d-galactosamine-induced hepatotoxicity in rats. J Ginseng Res. 39:376–383. 26869831 10.1016/j.jgr.2015.04.002 PMC4593786

[CIT0031] Lee SB, Kang JW, Kim SJ, Ahn J, Kim J, Lee SM 2017 Afzelin ameliorates d-galactosamine and lipopolysaccharide-induced fulminant hepatic failure by modulating mitochondrial quality control and dynamics. Br J Pharmacol. 174:195–209. 27861739 10.1111/bph.13669 PMC5192940

[CIT0032] Li X, Liu HC, Yao QY, Xu BL, Zhang SC, Tu CT 2016 Quercetin protects mice from ConA-induced hepatitis by inhibiting HMGB1-TLR expression and down-regulating the nuclear factor Kappa B pathway. Inflammation. 39:96. 26267064 10.1007/s10753-015-0227-9

[CIT0033] Li J, Zhong LP, Zhu HB, Wang FZ 2017 The protective effect of cordycepin on d-galactosamine/lipopolysaccharide-induced acute liver injury. Med Inflam. 2017:1–3946706. 10.1155/2017/3946706 PMC5387844 28522898

[CIT0034] Liang JC, Tan ZH 2013 Advances in animal models of liver fibrosis induced by DMN. Shandong Med J. 53:80–84.

[CIT0035] Liao CC, Day YJ, Lee HC, Liou JT, Chou AH, Liu FC 2017 ERK signaling pathway plays a key role in baicalin protection against acetaminophen-induced liver injury. Am J Chin Med. 45:105–117. 28081632 10.1142/S0192415X17500082

[CIT0036] Liao H, Cai J, Zhang LF, Peng Y, Wu P, Xie T, Pan Q 2017 A novel acute lethal liver injury mouse model with visualization of NF-κB activity for treatment of severe acute liver injury. Am J Transl Res. 9:962–970. 28386325 PMC5375990

[CIT0037] Li JJ, Chen K, Li SN, Liu T, Wang F, Xia YJ, Lu J, Zhou YQ, Guo CY 2016 Pretreatment with fucoidan from *fucus* vesiculosus protected against ConA-induced acute liver injury by inhibiting both intrinsic and extrinsic apoptosis. PLoS One. 11:0152570. 10.1371/journal.pone.0152570 PMC4818100 27035150

[CIT0038] Li F, Fan Z, Fang H, Gastroenterology DO 2015 The protective effect of *Hypericum perforatum* extract on acute liver injury in mice. Acta Univ Med Anhui. 50:477–481.

[CIT0039] Liu YW, Chiu YT, Fu SL, Huang YT 2015 Osthole ameliorates hepatic fibrosis and inhibits hepatic stellate cell activation. J Biomed Sci. 22:63–72. 26231226 10.1186/s12929-015-0168-5 PMC4522080

[CIT0040] Liu GT 1991 Present status of hepatitis drugs and ETs perspective in society office of China Association for Sciences and Technology Research on Prevention and Therapy of Viral Hepatitis. Chin SciTech Press. 16:337–341.

[CIT0041] Liu T, Xia YJ, Li JJ, Li SN, Feng J, Wu LW, Zhang R, Xu SZ, Cheng KR, Zhou YQ 2016 Shikonin attenuates concanavalin A-induced acute liver injury in mice via inhibition of the JNK pathway. Med Inflam. 1:2748367. 10.1155/2016/2748367 PMC4884842 27293314

[CIT0042] Li JJ, Xia YJ, Liu T, Wang JS, Dai WQ, Wang F, Zheng YY, Chen K, Li SN, Abudumijiti H 2015 Protective effects of astaxanthin on ConA-induced autoimmune hepatitis by the JNK/p-JNK pathway-mediated inhibition of autophagy and apoptosis. PLoS One. 10:1–20. 10.1371/journal.pone.0120440 PMC4356569 25761053

[CIT0043] Lu YL, Chen JW, Ren DY, Yang XB, Zhao Y 2017 Hepatoprotective effects of phloretin against CCl_4_-induced liver injury in mice. Food Agri Immunol. 28:211–222.

[CIT0044] Lu Y, Wang WJ, Song YZ, Liang ZQ 2014 The protective mechanism of schisandrin A in d-galactosamine-induced acute liver injury through activation of autophagy. Pharm Biol. 52:1302–1307. 24992201 10.3109/13880209.2014.890232

[CIT0045] Mahmoud MF, Hamdan DI, Wink M, El-Shazly AM 2014 Hepatoprotective effect of limonin, a natural limonoid from the seed of *Citrus aurantium* var. *bigaradia*, on d-galactosamine-induced liver injury in rats. Naunyn Schmiedebergs Arch Pharmacol. 387:251–261. 24258286 10.1007/s00210-013-0937-1

[CIT0047] Neyrinck A, Etxeberria U, Taminiau B, Daube G, Cani PD, Bindels LB, Delzenne NM 2015 A crude rhubarb extract protects from acute ethanol-induced liver damage in association with increased *Akkermansia muciniphila* population in the gut microbiota of mice. Clin Nutr. 34:S142–S143.

[CIT0048] Pan CW, Pan ZZ, Hu JJ, Chen WL, Zhou GY, Lin W, Jin LX, Xu CL 2016 Mangiferin alleviates lipopolysaccharide and d-galactosamine-induced acute liver injury by activating the Nrf2 pathway and inhibiting NLRP3 inflammasome activation. Eur J Pharmacol. 770:85–91. 26668000 10.1016/j.ejphar.2015.12.006

[CIT0049] Pan TL, Wang PW, Huang CH, Leu YL, Wu TH, Wu YR, You JS 2015 Herbal formula, *Scutellariae radix* and *Rhei rhizoma* attenuate dimethylnitrosamine-induced liver fibrosis in a rat model. Sci Rep. 5:11734–11744. 26133262 10.1038/srep11734 PMC4488958

[CIT0050] Parajuli DR, Zhao YZ, Jin H, Chi JH, Li SY, Kim YC, Sohn DH, Lee SH 2015 Anti-fibrotic effect of PF2401-SF, a standardized fraction of *Salvia miltiorrhiza*, in thioacetamide-induced experimental rats liver fibrosis. Arch Pharm Res. 38:549–555. 25005065 10.1007/s12272-014-0425-2

[CIT0051] Parameswari SA, Chetty CM, Chandrasekhar KB 2013 Hepatoprotective activity of *Ficus religiosa* leaves against isoniazid + rifampicin and paracetamol induced hepatotoxicity. Pharmacogn Res. 5:271–276. 10.4103/0974-8490.118828 PMC3807992 24174821

[CIT0052] Peng W, Qiu XQ, Shu ZH, Liu QC, Hu MB, Han T, Rahman K, Qin LP, Zheng CJ 2015 Hepatoprotective activity of total iridoid glycosides isolated from *Paederia scandens* (Lour.) Merr. var. *Tomentosa*. J Ethnopharmacol. 174:317–321. 26320683 10.1016/j.jep.2015.08.032

[CIT0053] Shih TY, Young TH, Lee HS, Hsieh CB, Hu OY 2013 Protective effects of kaempferol on isoniazid- and rifampicin-induced hepatotoxicity. Aaps J. 15:753–762. 23591749 10.1208/s12248-013-9490-6 PMC3691431

[CIT0054] Souli A, Sebai H, Chehimi L, Rtibi K, Tounsi H, Boubaker S, Sakly M, El-Benna J, Amri M 2015 Hepatoprotective effect of carob against acute ethanol-induced oxidative stress in rat. Toxicol Ind Health. 31:802–810. 23363576 10.1177/0748233713475506

[CIT0055] Song YF, Wang QK, He YH, Ren DD, Kow F, Li JW, Liu S, Cong HH 2017 The positive effects of fucoidans extracted from the brown seaweed *Saccharina japonica* on protection against CCl_4_-induced liver injury. J Appl Phycol. 29:2077–2087.

[CIT0056] Sun H, Zhang AH, Zou DX, Sun WJ, Wu XH, Wang XJ 2014 Metabolomics coupled with pattern recognition and pathway analysis on potential biomarkers in liver injury and hepatoprotective effects of yinchenhao. Appl Biochem Biotechnol. 173:857–869. 24728784 10.1007/s12010-014-0903-5

[CIT0057] Singh MP, Kim KY, Kim HY 2017 Methionine sulfoxide reductase a deficiency exacerbates acute liver injury induced by acetaminophen. Biochem Biophy Res Commun. 484:189–194. 10.1016/j.bbrc.2017.01.025 28104395

[CIT0058] Tailor A, Faulkner L, Naisbitt D, Park BK 2015 The chemical, genetic and immunological basis of idiosyncratic drug-induced liver injury. Hum Exp Toxicol. 34:1310–1317. 26614821 10.1177/0960327115606529

[CIT0059] Tang XW, Yang QL, Yang F, Gong JT, Han H, Yang L, Wang ZT 2016 Target profiling analyses of bile acids in the evaluation of hepatoprotective effect of gentiopicroside on ANIT-induced cholestatic liver injury in mice. J Ethnopharmacol. 194:63–71. 27582267 10.1016/j.jep.2016.08.049

[CIT0060] Tan H, He Q, Li R, Lei F, Lei X 2016 Trillin reduces liver chronic inflammation and fibrosis in carbon tetrachloride (CCl_4_) induced liver injury in mice. Immunol Invest. 45:371–382. 27219527 10.3109/08820139.2015.1137935

[CIT0061] Tang D, Wang F, Tang J, Mao A, Liao S, Wang Q 2017 *Dicranostiga leptopodu* (Maxim.) Fedde extracts attenuated CCl_4_-induced acute liver damage in mice through increasing anti-oxidative enzyme activity to improve mitochondrial function. Biomed Pharmacother. 85:763–771. 27923690 10.1016/j.biopha.2016.11.097

[CIT0062] Tan Z, Luo M, Yang J, Cheng Y, Huang J, Lu C, Song D, Ye M, Dai M, Gonzalez FJ, et al. 2016 Chlorogenic acid inhibits cholestatic liver injury induced by α-naphthylisothiocyanate: involvement of STAT3 and NFκB signalling regulation. J Pharm Pharmacol. 68:1203–1213. 27367057 10.1111/jphp.12592 PMC6300992

[CIT0063] Tian Y, Li Z, Shen B, Zhang Q, Feng H 2017 Protective effects of morin on lipopolysaccharide/d-galactosamine-induced acute liver injury by inhibiting TLR4/NF-κB and activating Nrf2/HO-1 signaling pathways. Int Immunopharmacol. 45:148–155. 28213269 10.1016/j.intimp.2017.02.010

[CIT0064] Uchida NS, Silva-Filho SE, Aguiar RP, Wiirzler LAM, Cardia GFE, Cavalcante HAO, Silva-Comar F. M d S, Becker TCA, Silva EL, Bersani-Amado CA, et al. 2017 Protective effect of *Cymbopogon citratus* essential oil in experimental model of acetaminophen-induced liver injury. Am J Chin Med. 45:515–513. 28359199 10.1142/S0192415X17500318

[CIT0065] Urfi MK, Mujahid M, Rahman MA, Rahman MA 2018 The role of *Tamarix gallica* leaves extract in liver injury induced by rifampicin plus isoniazid in Sprague Dawley rats. J Dietary Suppl. 15:24–33. 10.1080/19390211.2017.1310783 28459346

[CIT0066] Vladimir-Knežević S, Cvijanović O, Blažeković B, Kindl M, Štefan MB, Domitrović R 2015 Hepatoprotective effects of *Micromeria croatica* ethanolic extract against CCl_4_-induced liver injury in mice. BMC Complement Altern Med. 15:12. 26174335 10.1186/s12906-015-0763-8 PMC4501215

[CIT0067] Wang JW, Chen XY, Yang HP, Tan MM, Tang XG, Huang MC, Lou ZH 2016 Effects of *Linderae radix* extracts on a rat model of alcoholic liver injury. Exp Ther Med. 11:2185–2192. 27313665 10.3892/etm.2016.3244 PMC4888052

[CIT0068] Wang Y, Jiang YM, Fan XM, Tan H, Zeng H, Wang YT, Chen P, Huang M, Bi HC 2015 Hepato-protective effect of resveratrol against acetaminophen-induced liver injury is associated with inhibition of CYP-mediated bioactivation and regulation of SIRT1–p53 signaling pathways. Toxicol Lett. 236:82–89. 25956474 10.1016/j.toxlet.2015.05.001

[CIT0069] Wang SG, Pal P, Robert C, Huang HQ, Ding WX 2016 A mechanistic review of cell death in alcohol-induced liver injury. Alcohol Clin Exp Res. 40:1215–1223. 27130888 10.1111/acer.13078 PMC5455778

[CIT0070] Wang K, Song Z, Wang H, Li Q, Cui Z, Zhang Y 2016 *Angelica sinensis* polysaccharide attenuates concanavalin A-induced liver injury in mice. Int Immunopharmacol. 31:140–148. 26741264 10.1016/j.intimp.2015.12.021

[CIT0071] Wang LY, Tu L, Zhang JP, Xu K, Qian W 2017 Stellate cell activation and imbalanced expression of TGF-β1/TGF-β3 in acute autoimmune liver lesions induced by ConA in mice. Biomed Res Int. 2017:1–12. 10.1155/2017/2540540 PMC5303577 28246592

[CIT0072] Wang Y, Wan Y, Ye G, Wang P, Xue XW, Wu GZ, Ye BP 2016 Hepatoprotective effects of adiporon against d-galactosamine-induced liver injury in mice. Eur J Pharm Sci. 93:123–131. 27516150 10.1016/j.ejps.2016.08.017

[CIT0073] Wang LL, Wu GX, Wu FH, Jiang N, Lin Y 2017 Geniposide attenuates ANIT-induced cholestasis through regulation of transporters and enzymes involved in bile acids homeostasis in rats. J Ethnopharmacol. 196:178–185. 27988401 10.1016/j.jep.2016.12.022

[CIT0074] Wang CH, Gen QG, Wang YX 2012 Protective effect of atractylenolide I on immunological liver injury. J Chin Materia Medica. 37:1809–1813. 22997829

[CIT0075] Wang J-H, Wang J, Choi M-K, Gao F, Lee D-S, Han J-M, Son C-G 2013 Hepatoprotective effect of *Amomum xanthoides* against dimethylnitrosamine-induced sub-chronic liver injury in a rat model. Pharm Biol. 51:930–935. 23570518 10.3109/13880209.2013.770040

[CIT0076] Weerawatanakorn M, Hsieh S-C, Tsai M-L, Lai C-S, Wu L-M, Badmaev V, Ho C-T, Pan M-H 2014 Inhibitory effect of tetrahydrocurcumin on dimethylnitrosamine-induced liver fibrosis in rats. J Funct Foods. 7:305–313.

[CIT0077] Wei Y, Zeng B, Chen J, Cui G, Lu C, Wu W, Yang J, Wei H, Xue R, Bai L, et al. 2016 Enterogenous bacterial glycolipids are required for the generation of natural killer T cells mediated liver injury. Sci Rep. 6:36365. 27821872 10.1038/srep36365 PMC5099575

[CIT0078] Wu T, Chang MJ, Xu YJ, Li XP, Du G, Liu D 2013 Protective effect of *Calculus bovis sativus* on intrahepatic cholestasis in rats induced by α-naphthylisothiocyanate. Am J Chin Med. 41:1393–1405. 24228608 10.1142/S0192415X13500936

[CIT0079] Wu H, Qiu Y, Shu Z, Zhang X, Li R, Liu S, Chen L, Liu H, Chen N 2016 Protective effect of *Trillium tschonoskii* saponin on CCl_4_-induced acute liver injury of rats through apoptosis inhibition. Can J Physiol Pharmacol. 94:1291–1297. 27598942 10.1139/cjpp-2016-0228

[CIT0080] Wu MH, Qiu MY, Shu MZ, Zhang X, Li R, Liu S, Chen L, Liu H, Chen N 2016 Protective effect of *Trillium tschonoskii* Maxim saponin on CCl_4_-induced acute liver injury of rats through apoptosis inhibition. Can J Physiol Pharmacol. 94:1291–1297. 27598942 10.1139/cjpp-2016-0228

[CIT0081] Wunjuntuk K, Kettawan A, Charoenkiatkul S, Rungruang T 2016 Parboiled germinated brown rice protects against CCl_4_-induced oxidative stress and liver injury in rats. J Med Food. 19:15–23. 26075965 10.1089/jmf.2015.3460

[CIT0082] Xie W, Chen C, Jiang Z, Wang J, Melzig MF, Zhang X 2015 *Apocynum venetum* attenuates acetaminophen-induced liver injury in mice. Am J Chin Med. 43:457–476. 25967663 10.1142/S0192415X15500299

[CIT0083] Xie WY, Jiang ZH, Wang J, Zhang XY, Melzig MF 2016 Protective effect of hyperoside against acetaminophen (APAP) induced liver injury through enhancement of APAP clearance. Chemico-Biol Interact. 246:11–19. 10.1016/j.cbi.2016.01.004 26772156

[CIT0084] Xin X, Yang W, Yasen M, Zhao H, Aisa H. a 2014 The mechanism of hepatoprotective effect of sesquiterpene rich fraction from *Cichorum glandulosum* Boiss. et Huet on immune reaction-induced liver injury in mice. J Ethnopharmacol. 155:1068–1075. 24933227 10.1016/j.jep.2014.06.014

[CIT0085] Xu WX, Lu CF, Yao L, Zhang F, Shao J, Zheng S 2017 Dihydroartemisinin protects against alcoholic liver injury through alleviating hepatocyte steatosis in a farnesoid X receptor-dependent manner. Toxicol Appl Pharmacol. 315:23–34. 27939985 10.1016/j.taap.2016.12.001

[CIT0086] Yan D, Liu HL, Yu ZJ, Huang YH, Gao D, Hao H, Liao SS, Xu FY, Zhou XY 2016 BML-111 protected LPS/d-GalN-induced acute liver injury in rats. Int J Mol Sci. 17:1114–1127. 10.3390/ijms17071114 PMC4964489 27420055

[CIT0087] Yang YX, Han ZH, Wang YL, Wang LL, Pan S, Liang SW, Wang SM 2015 Plasma metabonomic analysis reveals the effects of salvianic acid on alleviating acute alcoholic liver damage. RSC Adv. 5:36732–36741.

[CIT0088] Yang QL, Yang F, Gong JT, Tang XW, Wang GY, Wang ZT, Yang L 2016 Sweroside ameliorates α-naphthylisothiocyanate-induced cholestatic liver injury in mice by regulating bile acids and suppressing pro-inflammatory responses. Acta Pharmacologica Sinica. 37:1218–1228. 27498779 10.1038/aps.2016.86 PMC5022106

[CIT0089] Yao XM, Li Y, Cheng XY, Li HW 2016 ER stress contributes to alpha-naphthyl isothiocyanate-induced liver injury with cholestasis in mice. Pathol-Res Pract. 212:560–567. 27173049 10.1016/j.prp.2016.05.001

[CIT0090] Zakaria ZA, Kamisan FH, Omar MH, Mahmood ND, Othman F, Abdul Hamid SS, Abdullah MNH 2017 Methanol extract of *Dicranopteris linearis* L. leaves impedes acetaminophen-induced liver intoxication partly by enhancing the endogenous antioxidant system. BMC Complement Altern Med. 17:271–285. 28521788 10.1186/s12906-017-1781-5 PMC5437572

[CIT0091] Zhang JJ, Meng X, Li Y, Zhou Y, Xu DP, Li S, Li HB 2017 Effects of melatonin on liver injuries and diseases. Int J Mol Sci. 18:673–700. 10.3390/ijms18040673 PMC5412268 28333073

[CIT0092] Zhang WX, Yin LH, Tao XF, Xu LN, Zheng LL, Han X, Xu YW, Wang CY, Peng JY 2016 Dioscin alleviates dimethylnitrosamine-induced acute liver injury through regulating apoptosis, oxidative stress and inflammation. Enviro Toxicol Pharmacol. 45:193–201. 10.1016/j.etap.2016.06.002 27317992

[CIT0093] Zhang JY, Zhang SM, Bi JB, Gu JX, Deng Y, Liu C 2017 Astaxanthin pretreatment attenuates acetaminophen-induced liver injury in mice. Int Immunopharmacol. 45:26–33. 28152447 10.1016/j.intimp.2017.01.028

[CIT0094] Zhang XK, Zhang L, Zhang HY, Cai ZY, Wang P 2016 Optimization extraction of *Crassostrea gigas* polysaccharides and its antioxidant activity and hepatoprotective against BCG-LPS-induced hepatic injury in mice. J Food Process Preserv. 40:1391–1399.

[CIT0095] Zhang N, Hu Y, Ding C, Zeng W, Shan W, Fan H, Zhao Y, Shi X, Gao L, Xu T, et al. 2017 Salvianolic acid B protects against chronic alcoholic liver injury via SIRT1-mediated inhibition of CRP and ChREBP in rats. Toxicol Lett. 267:1–10. 27989594 10.1016/j.toxlet.2016.12.010

[CIT0096] Zhang A, Jia Y, Xu Q, Wang C, Liu Q, Meng Q, Peng J, Sun H, Sun P, Huo X, et al. 2016 Dioscin protects against ANIT-induced cholestasis via regulating Oatps, Mrp2 and Bsep expression in rats. Toxicol App Pharmacol. 305:127–135. 10.1016/j.taap.2016.06.019 27317372

[CIT0097] Zhao YL, Wang JB, Zhou GD, Shan LM, Xiao XH 2009 Investigations of free anthraquinones from *Rhubarb* against alpha-naphthylisothiocyanate-induced cholestatic liver injury in rats. Basic Clin Pharmacol Toxicol. 104:463–469. 19389047 10.1111/j.1742-7843.2009.00389.x

[CIT0098] Zhao J, Zhang S, You S, Liu T, Xu F, Ji T, Gu Z 2017 Hepatoprotective effects of nicotiflorin from *Nymphaea candida* against concanavalin A-induced and d-galactosamine-induced liver injury in mice. Int J Mol Sci. 18:587–512. 10.3390/ijms18030587 PMC5372603 28282879

[CIT0099] Zheng W, Wang QL, Lu XH, Shi QQ, Zou JH, Tao YD, Wang P 2016 Protective effects of *Dracocephalum heterophyllum* in conA-induced acute hepatitis. Med Inflamm. 2016:1–8. 10.1155/2016/2684321 PMC4976189 27524863

[CIT0100] Zheng F, Sparkes A, De Baetselier P, Schoonooghe S, Stijlemans B, Muyldermans S, Flamand V, Van Ginderachter JA, Devoogdt N, Raes G, et al. 2017 Molecular imaging with kupffer cell-targeting nanobodies for diagnosis and prognosis in mouse models of liver pathogenesis. Mol Imaging Biol. 19:49–58. 27339464 10.1007/s11307-016-0976-3

[CIT0101] Zhou XL, Cheung CM, Yang JM, Or PMY, Lee WYW, Yeung JHK 2015 Danshen (*Salvia miltiorrhiza*) water extract inhibits paracetamol-induced toxicity in primary rat hepatocytes via reducing CYP2E1 activity and oxidative stress. J Pharm Pharmacol. 67:980–989. 25645193 10.1111/jphp.12381

[CIT0102] Zhou LT, Song YQ, Zhao J, Qin HY, Zhang GQ, Zhou Y, Wu XA 2016 Monoammonium glycyrrhizinate protects rifampicin- and isoniazid-induced hepatotoxicity via regulating the expression of transporter Mrp2, Ntcp, and Oatp1a4 in liver. Pharm Biol. 54:931–937. 26987268 10.3109/13880209.2015.1070878 PMC11132730

[CIT0103] Zhou X, Zhao HZ 2013 The experimental study of rhein on acute severe liver injury. J Clin Exp Med. 12:644–647.

